# The growing role of precision and personalized medicine for cancer treatment

**DOI:** 10.1142/S2339547818300020

**Published:** 2019-01-11

**Authors:** Paulina Krzyszczyk, Alison Acevedo, Erika J. Davidoff, Lauren M. Timmins, Ileana Marrero-Berrios, Misaal Patel, Corina White, Christopher Lowe, Joseph J. Sherba, Clara Hartmanshenn, Kate M. O’Neill, Max L. Balter, Zachary R. Fritz, Ioannis P. Androulakis, Rene S. Schloss, Martin L. Yarmush

**Affiliations:** 1 Department of Biomedical Engineering, Rutgers University, 599 Taylor Road, Piscataway, NJ 08854, USA.; 2 Department of Chemical & Biochemical Engineering, Rutgers University, 98 Brett Road, Piscataway, NJ 08854, USA.

**Keywords:** Cancer, Cancer Treatment, Precision Medicine, Personalized Medicine

## Abstract

Cancer is a devastating disease that takes the lives of hundreds of thousands of people every year. Due to disease heterogeneity, standard treatments, such as chemotherapy or radiation, are effective in only a subset of the patient population. Tumors can have different underlying genetic causes and may express different proteins in one patient versus another. This inherent variability of cancer lends itself to the growing field of precision and personalized medicine (PPM). There are many ongoing efforts to acquire PPM data in order to characterize molecular differences between tumors. Some PPM products are already available to link these differences to an effective drug. It is clear that PPM cancer treatments can result in immense patient benefits, and companies and regulatory agencies have begun to recognize this. However, broader changes to the healthcare and insurance systems must be addressed if PPM is to become part of standard cancer care.

## INTRODUCTION

### The complexity of cancer and its treatment

Cancer is one of the leading causes of death in the United States. In 2018 alone, there will be an estimated 1,735,350 new diagnoses and 609,640 cancer-related deaths^1^. Much work is ongoing to better understand and treat this group of diseases. The general defining feature of cancer is accumulated cell mutation, which manifests as tumors with uncontrolled growth. However, cancer is a complex, extremely heterogeneous condition. There are over 100 types of cancers, located in different organs and subtissues and originating from different cell types^2^. Some cancer types (e.g., colon, breast, and non-Hodgkin’s lymphoma) contain even more specific classifications based on their molecular subtypes^3–6^. Additionally, expression of markers within the same tumor can change depending on the specific location or stage of cancer. Despite this complexity and variability, most types of cancer are treated with the same generic therapies.

There are four main types of standard cancer treatments: surgery, radiation therapy, chemotherapy, and immunotherapy^7^. Some individuals will only require one treatment, but most often, a combination of treatments is used to tackle the resistant nature of cancer. Surgery can be used when there are solid tumors that have not metastasized and are located in accessible areas of the body; however, many cancers do metastasize, so more aggressive treatments, such as radiotherapy and chemotherapy, are needed. These approaches involve high doses of radiation and drugs in order to kill cancer cells and shrink tumors and, unfortunately, often cause additional damage to healthy cells. A study performed in 2004 estimated that the contribution of chemotherapy to overall survival in the United States was only 4.3%, due to chemotherapy drugs’ limited specificity^8^. Despite this, chemotherapy has been the standard of care in treating many different types of cancers, and oftentimes may be the only treatment that a patient receives. This low efficacy is not limited to only chemotherapy, but to other current cancer treatments as well — in fact, it is estimated that any particular class of cancer drugs is ineffective in a startling 75% of patients^9^. Notably, the effectiveness of these treatments depends on many individual factors, such as the type, stage, and location of the cancer as well as the patient’s age and overall health. This suggests that several personal factors should be considered before selecting a cancer treatment.

Another class of cancer treatments that have paved the way to more specific and effective therapies is immunotherapy, which harnesses a patients’ own immune system to fight cancer. Immunotherapy treatments include monoclonal antibodies (mAbs), checkpoint inhibitors, cytokines, vaccines, and adoptive cell transfer, most prominently in the form of hematopoietic stem cell transplants (HSCTs) and chimeric antigen receptor (CAR) T-cell therapies^10^. Adoption of immunotherapy has steered the field of cancer treatment toward the concept of precision and personalized medicine (PPM), in which therapy selection is tailored to each individual.

**Figure 1:**
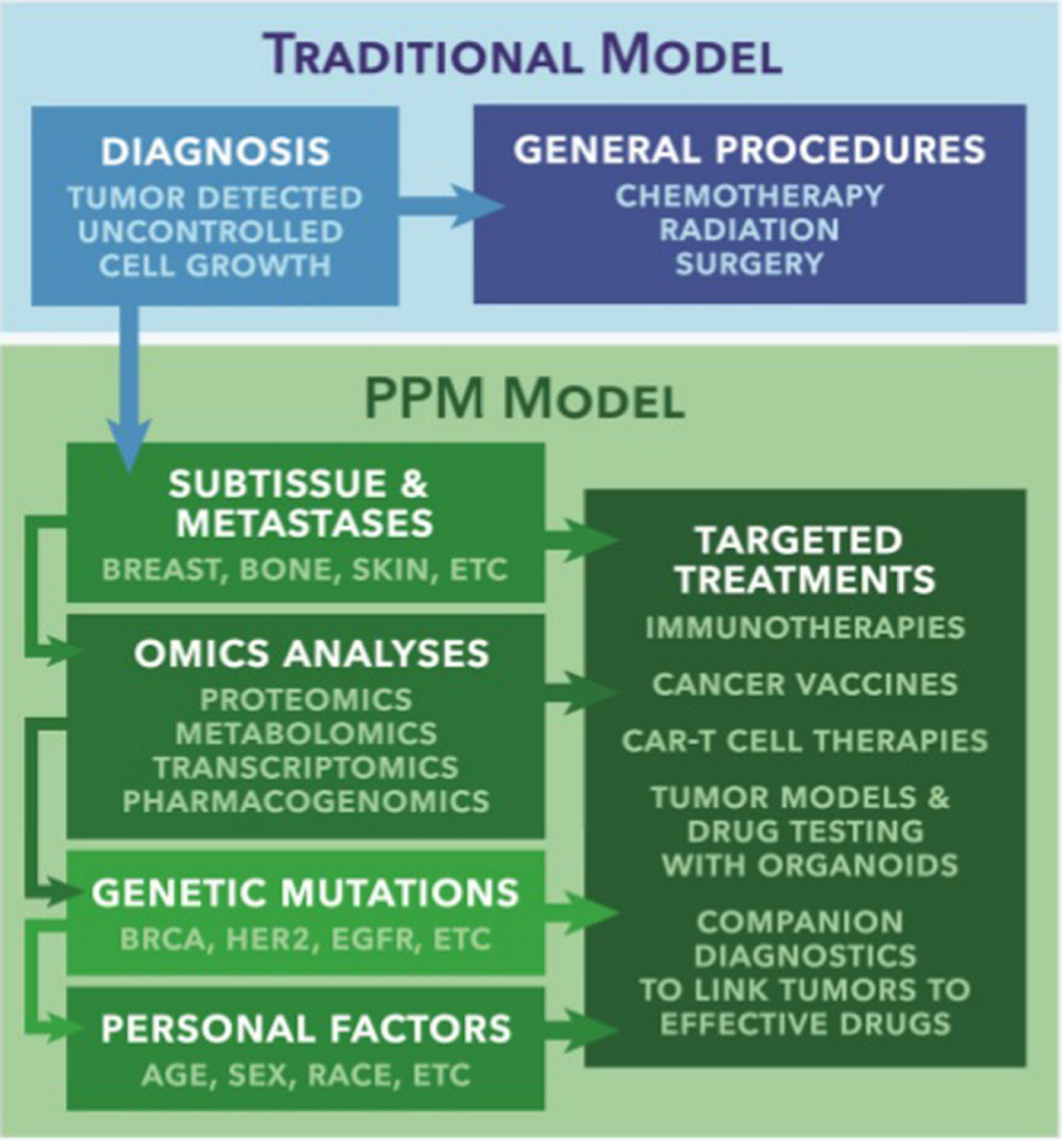
Traditional versus PPM model for cancer treatment. A comparison of the key differences in the traditional model of cancer treatment and the emerging precision and personalized medicine (PPM) model. Traditionally, cancer has been treated using general, “one size fits all” approaches such as chemotherapy, radiation, and surgical excision of tumors. These treatments vary widely in efficacy across individuals and also often cause harm to healthy, noncancerous organs and tissues. The PPM approach is characterized by individualized treatments tailored to specific tissues, gene mutations, and personal factors relevant to each unique case of cancer. Companion diagnostics (CDx) help identify which treatments will be most effective for a specific patient’s tumor, and novel cell therapies are used to target the cancer with minimal damage to healthy tissues, making the PPM model more effective and safer.

Over the past decade it has become increasingly clear that no two patients’ cancers are exactly the same, and hence, may have variable responses to generic treatments such as chemotherapy and radiation^11^. This traditional model for cancer therapy is overly simplified; it results in ineffective, expensive treatments and causes patients to suffer from unnecessary side effects. A more effective model, poised to change this “one size fi ts all” approach, is based on PPM^12^ (Fig. 1). This perspective fosters the development of specialized treatments for each specific subtype of cancer, based on the measurement and manipulation of key patient genetic and omic data (transcriptomics, metabolomics, proteomics, etc.). For example, Soda *et al.* identified a mutation in the anaplastic lymphoma kinase (ALK) that drives tumor formation in about 5% of non-small-cell lung cancers^13^. This discovery led to the development of ALK blockers such as crizotinib and ceritinib, Food and Drug Administration (FDA) approved drugs given to patients who test positive for the ALK mutation. A similar example is the promising use of the poly ADP ribose polymerase inhibitor olaparib in the treatment of BRCA-mutant ovarian cancer^14^.

There also exists a growing category of PPM products called companion diagnostics (CDx), which are molecular assays that measure levels of proteins, genes, or specific mutations to reveal a specific, efficacious therapy for an individual’s condition^15^. Some examples include Dako Denmark’s HERCEPTEST and HER2 FISH PharmDx Kit, which determine HER2 protein and gene overexpression in fixed breast, metastatic gastric, or gastroesophageal junction adenocarcinoma tissues^16^. Another example, Myriad Genetic Labs’ BRACAnalysis CDx, detects and classifies DNA variants in the protein coding region of the BRCA1/2 genes using patient whole blood samples^17^. These CDx allow for the selection of a treatment that is more likely to be effective for each individual based on the specific characteristics that their cancer possesses. The FDA has shown support in the PPM approach with their approval of these and other technologies since 1998, when the drug trastuzumab was approved for the treatment of HER2 receptor positive breast cancer^18^. Furthermore, the enactment of the Precision Medicine Initiative in 2015 has also pushed the PPM field forward, by requiring the FDA to develop new platforms to evaluate PPM diagnostics and therapies^19^.

It is clear that integrating a PPM perspective into cancer research and treatment could result in major improvements in fighting cancer, especially due to its complexity and interpatient variability. In the current state of science and medicine, this has already started to be recognized through PPM research, PPM products and support from the FDA; however, there are several broader, societal obstacles that must be addressed and overcome before PPM can become fully integrated into standardized care.

### The PPM process and integration into cancer treatment

The field of PPM is designed to develop therapies for a single subject or subject group based on data that captures current and past physical health and environmental exposure. Based on these data, patients are categorized into groups for different, clinically relevant purposes. A few examples of the uses of PPM include determining genetic predisposition to a disease, identifying patient groups for clinical trials, and identifying individuals that are more likely to respond well to a specific therapy.

The completion of the Human Genome Project (HGP) gave scientists the ability to read and interpret an individual’s genetic code and to identify genetic predispositions to certain diseases. This milestone event changed the perspective on health from reactive to preventative. Today, scientists are working toward obtaining a detailed understanding of the function of the body from multiple omics levels and characterizing how genetic predispositions are affected by environmental exposures. Taken together, all of this information will ultimately allow scientists and doctors to better predict how patients will respond to a certain treatment. As highly valuable tools that assist personalized therapies, CDx assay patients for genetic traits that identify whether the patient would respond to a particular treatment. This approach can have a major impact on the care of the patient. The revolution lies in the change from a clinician selecting a generic therapy that is more or less experimental for the patient, to one that effectively targets the disease with PPM.

This review comments on the fields of *personalized medicine* **and** *precision medicine*, taken together as PPM. Although today the terms are often interchanged — they both refer to the use of unique characteristics from patients to select the best treatment — the field was originally referred to as *personalized medicine*^20^. However, as it gained popularity and the term became more widely used in science, media, and society, it began to carry a misconception. Many people incorrectly assumed that due to the “*personalized*” nature, unique treatments were being developed for each individual. In order to clarify the actual goal of the field, the scientific community, specifically the National Research Council, has pushed for the use of *precision medicine* to replace the misleading name of *personalized medicine*^21^. Still, *personalized medicine* continues to be more widely recognized by the general public. We consider both terms in the current review in order to be inclusive of both perspectives throughout recent history and to acknowledge the evolution of the terminology.

**Figure 2:**
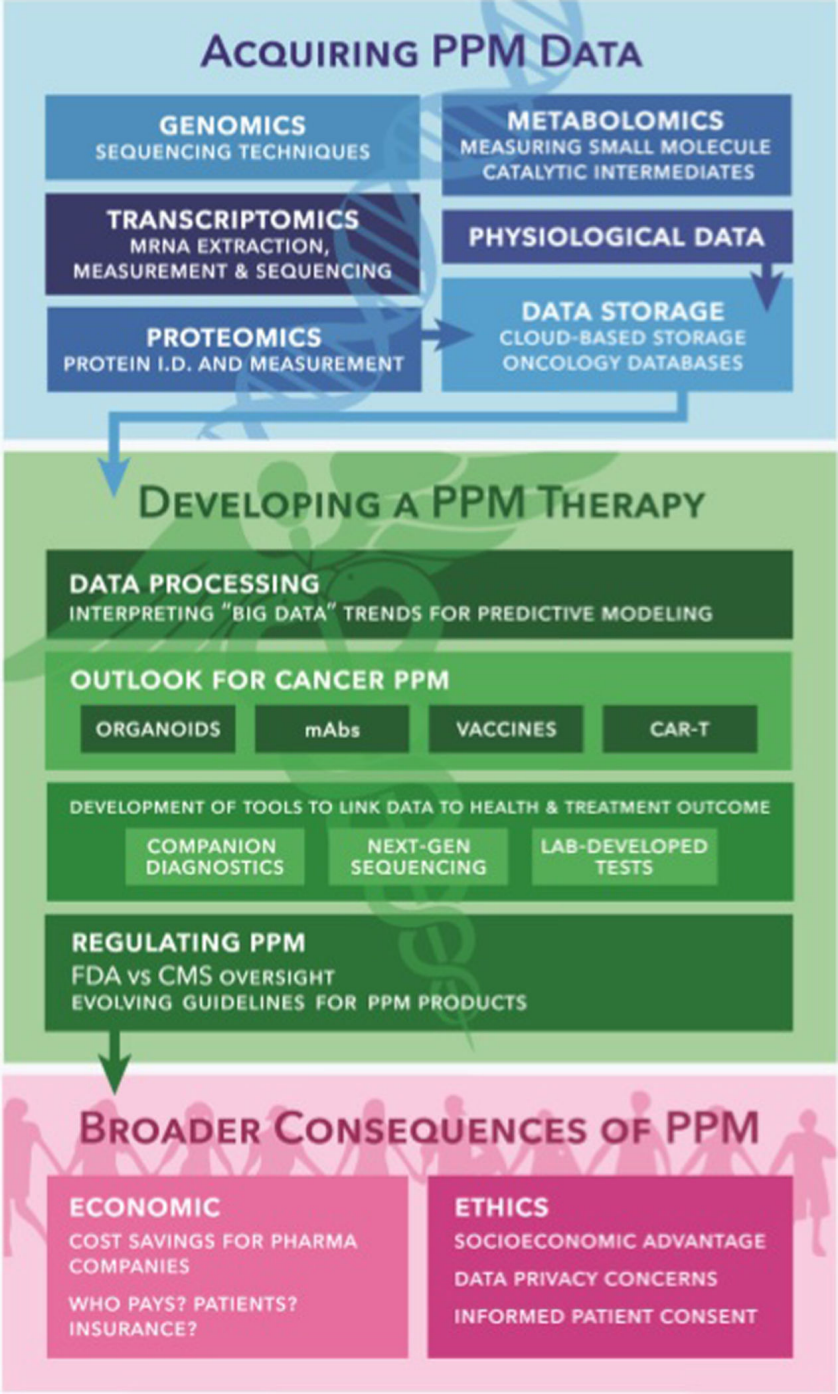
The PPM process: From data acquisition to integration in healthcare. A flowchart of the general process of PPM treatment, which serves as an outline for this article. First, a large volume of “omics” data is acquired from the patient and stored in one of several cloud-based databases. We discuss the various technologies that allow for omics data acquisition. Data processing algorithms identify the unique features of the patient’s cancer, and companion diagnostics (CDx) tools, which we discuss next, link these features to specific treatments that will likely be the most effective at treating the cancer. We outline the development of several of these products, including targeted antibodies, cancer vaccines, and T-cell therapies. The regulation of new PPM treatments and products by the Food and Drug Administration (FDA) and Center for Medicare and Medicaid Services (CMS) is continually evolving; we discuss the landmark regulatory changes that have enabled approval of new technologies and consider the future of the regulatory landscape. Finally, we look at the economics and ethics of PPM, including how to reduce cost, who to hold responsible for payments, and concerns about accessibility and data security.

In this review, the current state of the field of PPM in regards to cancer is presented in three categories, which are depicted in the flowchart in Fig. 2. We begin by describing the methods of **(1) Acquiring PPM Data**. Here, the multiple omics techniques (genomics, transcriptomics, proteomics, and metabolomics) used to characterize an individual’s disease state are discussed. The understanding and application of these data as tools in clinical trial design and treatment selection are discussed in, **(2) Developing a PPM Therapy**. Emerging cancer products, such as organoids, mAbs, cancer vaccines, and CAR T-cells are also presented from a PPM perspective. Also addressed are the evolving federal regulations for PPM products, in order to ensure their safety and efficacy. In **(3) Broader Consequences of PPM**, the economic and ethical concerns of PPM are considered. Establishing PPM is complicated from an economic point-of-view, likely requiring alterations to the contemporary insurance-payer system. The nature of the field can also be daunting from an ethical perspective, requiring the establishment of sufficient protections to the privacy and health of targeted patients.

It is the opinion of this review that the field of PPM is beneficial to the patient and the scientific community, by stretching collaborations and expanding understanding of the biological complexity of cancer and its treatments. This, however, does not come without the broad challenges and adaptations that are associated with newly emerging fields, particularly from the standpoint of biotechnology companies and society as a whole.

## ACQUIRING PPM DATA

Before a PPM treatment can be developed and used in patients, a specific gene or mutation must be correlated with a clinical outcome. This is a major undertaking; it can take years of research performed by many scientists to uncover a phenotype or polymorphism that is clinically meaningful. Furthermore, understanding which polymorphism leads to a positive versus negative treatment response in patients requires additional analysis. The first step in this process toward understanding the genetic code is to sequence DNA from many individuals. With the advancement of sequencing technologies, this step is becoming easier. The major challenges lie in interpretation of these enormous data sets, which is where bioinformatics plays a major role.

### Genomic sequencing technologies

The field of PPM would not exist without the major accomplishment of sequencing the human genome. The HGP took 13 years to complete, from 1990 to 2003. This was a major undertaking by the International Human Genome Sequencing Consortium (IHGSC), consisting of over 200 collaborating labs in 19 countries, discovering new information about the structure and organization of the genome^22^. It was discovered that there are approximately 20,500 genes within the human genome and that any two individuals share 99.99% of their genome, indicating that genetic individuality could be identified within only the remaining 0.01%. Furthermore, long repeat sequences were identified within the genome, and differences in single bases (single nucleotide polymorphisms [SNPs]) held the potential to be unique disease indicators^22^. This initial information gathering was facilitated by two methods through the use of bacterial artificial chromosomes (BAC) and Sanger sequencing. BAC vectors facilitated the initial phase of genome sequencing, functioning to determine the chromosomal location of DNA fragments isolated from a sample^22^. In contrast, Sanger sequencing enabled the precise base-by-base identification of a DNA fragment^22^. Although essential in early sequencing efforts, these methods were expensive and inefficient. As a result of several years of research and development to overcome these problems, Next Generation Sequencing Technologies (NGSTs)^23^ have emerged. NGSTs expand upon the BAC and Sanger sequencing methods, providing cost-effective tools capable of high-dimensional and parallel sequencing^23^. Table 1 details several currently available NGSTs along with their advantages and disadvantages.

With today’s technology, the scientific community can sequence genetic information with relative ease. Current challenges involve correlating genetic details with predisposition to disease. Similarly, the genome is not an exclusive variable in a patient’s state of health. Other omics levels, requiring other forms of technology beyond DNA sequencing, provide insight into a subject’s health via measurement of protein structure and function, epigenetic manifestations, the mechanisms of metabolism, and the concentration of metabolic intermediates^24^.

### Transcriptomic, proteomic, and metabolomic techniques

While genomic data is critical to developing a comprehensive understanding of disease progression and drug effects in physiological systems, bridging the gap from genotypic effect to phenotypic event is accomplished by characterizing intermediate omics levels, including the transcriptome, proteome, and metabolome.

#### Transcriptomics

The total mRNA within a subject or sample is defined as the transcriptome^25,26^. Contemporary high-throughput sequencing techniques for collecting transcriptomic information include microarray and RNA sequencing (RNA-Seq) methods (Table 2). Microarray analysis identifies mRNA expression by measuring the level of hybridization between a sample and complementary probes. The abundance of gene expression within a sample is indicated by the level of fluorescence found within each well of the array corresponding to a particular probe^25^. Microarray analysis is limited in that prior knowledge of the gene’s sequence is required to design probes^25^. Distinct from microarray analysis, RNA-Seq is useful for measuring mRNA expression level as well as discovering new sequences, as this process does not require probes or prior knowledge of the mRNA sequence of interest^25,26^. This method is analogous to Sanger sequencing, in that the mRNA sequence is determined by the one-by-one addition of fluorescently-labeled nucleotide bases. Fluorescent images are captured during each iteration, and their analysis reveals the specific sequence, as well as its expression level^26^. Microarray analysis requires less labor preparing samples than does RNA-Seq^25^; however, RNA-Seq does not require prior knowledge of gene sequences and can process smaller quantities of samples^25,26^. Both methods have high throughput capabilities, though microarray currently possesses better cost-value^25^.

Contemporary drug development is enabled through genomic profiling, generally incorporating either microarray analysis or RNA-Seq for transcriptomic profiling. Both microarray and RNA-Seq analyses enable the characterization of disease phenotype and drug effect within a system (single-cell or larger), which provides invaluable information for the development of genome-specific therapies^27^. RNA-Seq appears advantageous for the discovery of novel genomic drug effects and disease phenotypes; however, microarray analyses are cheaper and have more standardized protocols^25,27^. In general, RNA-Seq is more advantageous for clinical investigations because it is capable of delivering a lower signal-to-noise ratio than microarray results. Furthermore, RNA-Seq results can be acquired from smaller sample quantities compared to microarray methods — nanogram versus microgram masses, respectively^25,27^. It is predicted that as NGSTs become more integrated in clinical diagnostics, RNA-Seq protocols will become more standardized and replace microarray diagnostics^27^. Currently, both diagnostic tools are used to generate transcriptomic results depending on financial and experimental necessity^27^.

#### Proteomics

Proteomics refers to identifying and cataloguing all proteins, and the interactions between these proteins, in a cellular system. Proteomic measurements yield information about protein structure, concentrations and cellular localizations, protein–protein interactions, and protein synthesis and degradation rates. This information is used to understand how the proteome changes during different biological processes and for identifying patterns of disease^28^. For PPM, data on post-transcriptional modifications, or abundance of proteins in a tissue, could be important for disease diagnosis, progression and treatment. Over the past two decades, mass spectrometry (MS) has been the main tool used for collecting proteomic data, particularly to measure protein expression, identify sites of protein modification, and investigate protein–protein interactions^29^.

Two major strategies have evolved to generate proteomic data: bottom-up and top-down proteomics. These methods and other subcategories are summarized in Table 3. The bottom-up strategy, also known as “shotgun proteomics,” uses MS to analyze large mixed protein samples and determine their composition. Generally, the bottom-up strategy is useful for analyzing an unknown mixture of proteins but is imprecise for several reasons: information about a particular protein can be lost when it is fragmented, MS data can be easily misinterpreted, and only proteins with high concentrations in the mixture appear on the MS output. Still, the shotgun approach is useful in PPM as it enables the generation of a unique proteomic “fingerprint” for each patient. This can result in the identification of key protein biomarkers for particular disease states^30^. Recent labeling technologies have enabled simultaneous multiple-sample shotgun analyses (bottom-up labeling), which additionally facilitates examination of proteomic changes due to biological perturbations.

The top-down strategy is a newer approach and involves MS analysis of whole proteins, after which a particular protein of interest can be isolated, fragmented, and further analyzed. Top-down proteomics is a critical tool for studying post-translational modifications of proteins, which helps elucidate protein function. Post-translational modifications are often linked to disease states, particularly in cancer, diabetes, infectious and neurodegenerative diseases, and blood disorders^31^. In the context of PPM, identifying key post-translational modifications in individual patients could prove a potent diagnostic tool. Additionally, analyzing temporal expression of a particular protein could provide clinicians with detailed pharmacodynamic information about therapeutic drugs^30^.

A hybrid strategy, termed “middle-down proteomics,” has emerged in recent years as an attempt to optimize the advantages of both techniques. Like bottom-down proteomics, middle-down proteomics uses protein digestion but seeks to produce significantly larger peptides, thus producing less complex and ambiguous protein solutions and also enabling analysis of high-level characteristics^30^. Middle-down proteomics has already been established as the best method for studying histone proteins^32^. It also shows promise for PPM applications as it allows for both quantification of a large number of potential protein biomarkers and analysis of individual protein mutations and modifications.

#### Metabolomics

Metabolites are the small-molecule intermediate products in metabolic reactions, and metabolomics refers to their identification and analysis. Metabolites are useful because they reflect both genetic and environmental influences, and a complete metabolic analysis is often described as a “functional readout” of the current state of the organic system^33^. In a PPM context, metabolomic data could offer insight into an individual’s unique physical reaction to a drug, an application that is also referred to as metabolomics^34,35^. At present, metabolomic studies of biofluids and tissues have contributed to the development of PPM approaches by identifying biomarkers for disease states, which have the potential to assist clinicians in diagnosis and early treatment^36^. One of metabolomics’ key clinical advantages is that measurements can be made noninvasively, since metabolites, unlike most proteins, diffuse throughout the body and appear in easily accessible biofluids, like blood and urine^33^. In the early days of metabolomics studies, nuclear magnetic resonance (NMR) spectroscopy was often used to identify metabolites, but the past decade has seen a major shift toward MS, which offers higher resolution and sensitivity to small concentrations^37^.

Like in proteomics, metabolomic strategies (Table 4) can be broadly classifi ed into two categories: targeted and untargeted approaches. The untargeted, or global, approach has been used to characterize the metabolomic fingerprint in a variety of diseases, including Parkinson’s disease, Crohn’s disease, diabetes, liver disease, and multiple forms of cancer^33,38^. Due to the wide range of metabolite concentrations in a standard sample — estimated to cover 7–9 orders of magnitude, from pmol to mmol — there is no single technology that can provide a complete fingerprint of all metabolites^39^. However, improvements in liquid and gas chromatography technologies have enabled cleaner metabolite separations, while advancements in MS resolution have allowed for the detection of large numbers of distinct peaks at multiple concentration levels^40^. Accurately identifying the thousands of peaks generated by an untargeted experiment continues to be the greatest challenge associated with this technique, and high false positive rates pose issues for clinical adaptation in PPM^38,41^. Nevertheless, untargeted metabolomics is a critical technique for generating hypotheses about potential biomarkers.

Targeted metabolomics aims to quantify known metabolites in a particular sample and represents the bulk of metabolomics research in PPM. The targeted approach enables clinicians to measure samples of a patient’s biofluids for anomalous metabolite levels that could lead to a diagnosis. Alternatively, clinicians can use this technique to monitor metabolic responses following administration of a drug, in order to determine an exact dosage regimen. However, in order for this technique to have clinical relevance, identification and rigorous confirmation of appropriate metabolic biomarkers must be completed. Metabolomics is a promising tool for the advancement of PPM, especially when used in conjunction with other omics data. This is a venture that requires specialized bioinformatics tools. The field is beginning to see the emergence of robust tools for omics integration — including Metabox, a free R-based application that combines metabolomic, proteomic, and transcriptomic data^42^, and MKGI models, which use neural networks to identify interactions between different omics data sets^43^. These are just a few examples of the many integrative tools available^44^, which are key to bringing omics approaches to the clinic.

### Physiological and lifestyle data

A patient’s physiological and lifestyle factors are also important, as one’s habits influence disease progression and response to treatment^45^. Many clinical studies have demonstrated the impact of physiology on the absorption, distribution, metabolism, and elimination (ADME) of drugs in the body^46–48^. Physiological differences due to age, sex, ethnicity, and stage of disease have been shown to affect pharmacokinetic response to drugs as well as increase the variation in responses^45^. For example, drug clearance tends to be lower in geriatric patients compared to young adults. This can lead to significant dissimilarities in drug elimination behavior, thus resulting in different pharmacokinetic responses, quantified in the form of bioavailability^49^. Similarly, lifestyle and environmental conditions have long been shown to have a strong effect on disease^50^. Healthy diets and moderate exercise are generally affiliated with lower risks for disease, whereas lifestyle choices such as excessive smoking and alcohol consumption have been linked with cancer and other diseases^51^.

One of the challenges with advancing PPM based on physiological information is associated with the lack of available anatomical data characterizing specific differences between broader subpopulations of patients such as age, gender, ethnicity, and disease. This, in part, is due to the high variations that exist even within these subpopulations^52^. In contrast, the availability of data for PPM based on omics is plentiful. Improved technologies have enabled the availability of tremendous volumes of data, but the information they provide is complex^53^. The challenge there lies in properly storing, analyzing, interpreting, and utilizing these data so that they can reach their full, clinical potential.

### Data storage

While omics data offer great potential for understanding disease, their acquisition also presents a major challenge: storage. The National Cancer Institute’s Cancer Genome Atlas contains 2.5 petabytes of data, which is equivalent to over 530,000 DVDs^54^. Additionally, by 2013, over 20 petabytes of data related to genes, proteins, and small molecules were recorded by the European Bioinformatics Institute^55^. While the generation of such large volumes of data is common practice for fields like high-energy physics and astronomy, it is a relatively uncharted territory for biology^55^.

Effective use of omics data relies on appropriate data storage and accessibility for researchers and clinicians. Companies such as Amazon supply cloud computing resources that have improved data storage capabilities for PPM. The Amazon Web Services provides a cloud-based data storing platform used by organizations like the National Institute of Health’s Human Microbiome Project (HMP), the INOVA Translational Medicine Institute (ITMI), and GenomeNext^56^. Highly curated databases are essential to improving data analysis for PPM^57^. Database developers must provide user-friendly interfaces in order to efficiently provide the full availability of data to researchers. Databases must also be refined in an iterative manner as new information becomes available to ensure recent and updated content.

A variety of massive databases exist for oncology data, notably the International Cancer Genome Consortium (ICGC) and The Cancer Genome Atlas (TCGA; run by the National Cancer Institute and the National Human Genome Research Institute)^58^. Information within ICGC’s data portal focuses on 50 tumor types and characterizes them on genomic, transcriptomic, and epigenomic levels across genders, mutations, tumor stage, and more. The TCGA portal provides detailed information on genetic mutations and gene expression in 11 types of cancer tissues, in a total of 33 subtypes of cancer^54^. Analysis is performed on high quality tumor samples and matched normal tissue samples, on a high quantity of patients^58^. Also worth mentioning is the Catalogue of Somatic Mutations in Cancer (COSMIC), which allows researchers to browse the database according to cancer types, tissue, and genes. Kyoto Encyclopedia of Genes and Genomes (KEGG) is another widely used genomic resource that links genomic data to systemic functions and reports most genes in the context of both function and molecular pathways^59^. These are just a few of many available cancer databases that aid in PPM research efforts^58^. Through effective use of omics methods and databases, key genes, metabolites, and proteins can be linked to a disease state. Therapies can then be identified or developed to effectively treat cancers on a personalized level.

## DEVELOPING A PPM THERAPY

Once biological data have been acquired and stored, they must be analyzed, with the goal of identifying biomarkers, mutations, or pathways relevant to disease or treatment outcome. The field of systems biology aids in these efforts by analyzing data from preclinical and clinical studies. Statistical and modeling techniques are used to identify and assess mechanistic relationships within biochemical systems. This analysis is used to develop predictive tools that replicate biological systems in order to characterize their behavior and response in the context of disease and drug development. This is particularly relevant to forthcoming cancer treatments, as approximately 73% of oncology drugs in development are personalized medicines^9^. PPM therapies that are currently being developed include cancer vaccines, mAbs, and CAR T-cells. Organoids are being used as *in vitro* models to understand tumor heterogeneity and the variability of patient response to cancer treatments. As these PPM products and services emerge, it is pertinent that companies are aware of the evolving regulatory landscape for the PPM field and continuously reference updated guidance documents.

### Linking omics data to treatment

A major challenge in PPM lies in establishing the relationship between biological data, disease, and clinical translation: how can we interpret the data collected to make meaningful medical decisions? “Big Data,” in reference to the medical industry, refers to the greater collection of medical data across thousands of patients, involving the tracking of various medical indicators and biomarkers (primarily clinical and omics data). High-throughput data collection enables researchers to screen tissues for thousands of molecular targets, effectively capturing the response of a complex system over time. Within the field of systems biology, reconciliation of these omics components enables the construction of predictive models of human physiology used in experimental design and clinical trial development^60–63^.

In order to correlate observations with biological events and phenotypes, systems biologists and bioinformatic scientists employ techniques to identify statistically significant trends^64^. These include multivariate decomposition techniques, predictive modeling and optimization techniques, and other statistics-based tools. Statistically interpreting trends from Big Data is a discipline unto itself and is necessary for predictive modeling and clinical decision support^65^.

It is important to remember that Big Data is a network of information that is both useful and deafening — analysts of which suffer the burdens of “missing values, curse of dimensionality, and bias control”^65^. Big Data is not an easy-to-use information source from which trends connecting diseases to patient characteristics can be simply identified. Instead, Big Data is a multidimensional network containing medical information from thousands of patients, all of whom are influenced by different environments, have unique genomes and epigenomes, and who are analyzed by different physicians prone to unique biases and varied techniques. Furthermore, patient screening, sample collection and analysis, and even physical measurements are all subject to bias. All of these considerations contribute to the complexity of the PPM field.

### Omics tests and clinical trials

Omics data is essential to the development of targeted therapies as well as patient stratification, notably within preclinical experiments and clinical trials. Le Tourneau *et al.* reviews specifications for establishing PPM clinical trials — those that select patients likely to respond to the experimental treatment, as determined by molecular profiling of tumors^66^. This field of study, called pharmacogenomics, uses experimental and quantitative sciences to analyze the influence of genomics on drug effects^67^. Diseases and drug effects are conventionally correlated to macro data, such as age, weight, and gender. Improvements in high-throughput screening technology and increasing reliance on computational tools have enabled the development of the pharmacogenomics field, which correlates drug effects with omics data while revealing gaps in which drug targeting can be made more specifi c^67^. Such pharmacogenomic analysis can ultimately identify patient populations likely to be responsive to targeted therapies, which is a primary goal of PPM.

**Figure 3:**
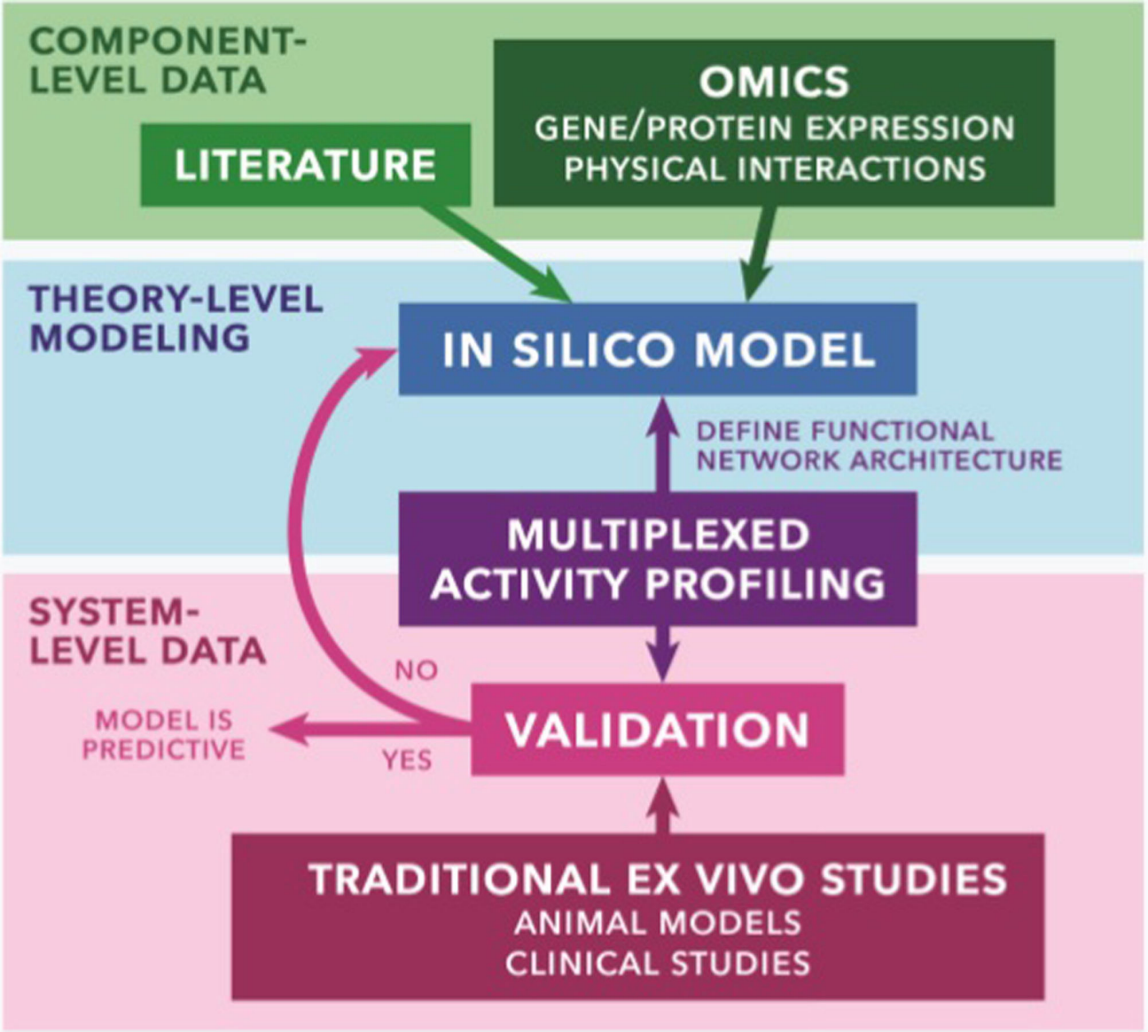
Predictive model development from large-scale omics data. An overview of the process for development of predictive models. Turning gigabytes of patient data into relevant clinical information requires a Big Data approach — specifically, predictive algorithms that are refined and validated with results from data-driven investigations, including traditional animal model studies and clinical trials. Adapted by permission from [**RightsLink Permissions Springer**]: [**Springer Nature] [NATURE BIOTECHNOLOGY]** Butcher *et al.*^60^, **[COPYRIGHT]** (2004).

To get from omics data to patient stratification in clinical trials, predictive computational models must be used. Here, each molecular target from the omics measurements is a variable in a complex system that represents the tissue^68–70^. Statistical techniques are applied in order to segregate noise from usable information and ultimately reveal physiological trends based off of key molecular markers. The resulting *in silico* model is further modified by data-driven investigations whose results are analyzed and fitted to mathematical models. Validation of these models is accomplished with additional training data. Once verified, the model is predictive and can be used in further experimental design or clinical trial guidance (Fig. 3)^60^.

The combination of omics assays and a specific computational model (omics predictor) is defined as an omics test^63^. There are two types of omics tests: prognostic test, which predicts a clinical outcome in the form of a measurement; and a therapy guiding test, which identifies subgroups of patients that are unique in their response to a particular therapy^63^. Notably within the realm of cancer research, omics tests are applied in identifying and validating biomarkers for disease indications^61^. Establishing the validity of the biomarker for a disease indication requires validation using an omics test, and this omics test (sample preparation, performing the sequencing assay, computational pipeline for assessing the sequence read) also requires validation^61^. Clinical viability and utility of the biomarker and omics test must be established, meaning that the use of this biomarker should result in the end-point of progression-free survival^61,71^.

Clinical trials examining the efficacy and utility of validating biomarkers with omics tests are not yet commonly successful (see the National Academy of Sciences report *Committee on the review of omics-based tests for predicting patient outcomes in clinical trials* for an extensive review^71^). In short, putative biomarker identification is expedited with omics analyses; however, establishing clinical validity and clinical utility is more difficult. A contemporary primary research focus is the effort to establish safe and effective use of omics tests in clinical trials^61–63^. More specifically, the development of novel and robust statistical analysis methods must undergo the same rigorous development as bioassays^63^. To enable this, the Institute of Medicine established guidelines for the use of omics analyses in clinical trials in 2013, which the U.S. National Cancer Institute (NCI) adheres to when reviewing proposals for studies involving omics tests^61,62^. In addition to providing recommendations for application of omics-based tests in clinical trials, these guidelines require that agencies receive FDA approval for all “sequencing assays and their associated analysis software tools as potential investigational devices … [and provide] public availability and transparency of raw data as a means to enable the external validation of omics-based trials”^61,62^. McShane *et al.* of the NCI also indicates that as researcher teams with greater variety of expertise (laboratory, computational, bioinformatics, and clinical) develop, omics tests will become more robust^63^. These institutes and contemporary researchers expect an increase in the number of successful clinical trials incorporating omics tests as expertise expands and rigor improves.

### Clinical outlook for PPM cancer products

Advancements in omics technologies have led to drug discovery approaches for a variety of PPM cancer products^72^. Detection methods for circulating tumor cells (CTCs) and DNA are promising not only for early diagnosis, but also for individualized patient risk monitoring and identification of effective personalized treatments. Another approach has focused on recapitulating individual tumors *in vitro*, in order to determine the safest and most effective treatment before administration to a patient. Several other therapies under development harness the unique power and specificity of the immune system to combat cancers. Over a century of work has focused on this and has evolved into a distinct discipline called immunoengineering. The ultimate goal of this field is to tailor an increasingly specific and potent immune response, which can result in a powerful, effective, and personalized cancer treatment^73^.

#### CTCs and DNA for early cancer detection

Two types of oncological biomarkers, CTCs and circulating tumor DNA (ctDNA), have emerged as the face of the “liquid biopsy” techniques focused on noninvasive cancer diagnostics. Research supporting the notion that tumors shed both types of biomarkers into the bloodstream early on in cancer progression has meant that much focus has been placed on their applications for early detection and screening^74–76^. As research continues and technology improves, CTCs and ctDNA are also likely to prove useful in risk stratification, disease monitoring, and personalized treatment selection.

The biggest challenge in implementing CTC detection techniques is the rarity of these biomarkers: estimates place CTC frequency at one cell per 10^6^–10^7^ leukocytes^77^. Thus, CTC detection techniques require some form of sample enrichment or isolation step, such as immunoaffinity/ antibody targeting of cell surface markers, size exclusion methods, or separation on the basis of electrical properties. These sample preparation steps are not without their own issues: CTC viability can be negatively affected by these processes, the heterogeneity inherent to CTCs means not all cells may be detected, and the lack of standardized protocols has resulted in significant variability in results between techniques, operators, and laboratories^77^. However, once captured, CTCs can provide a wealth of omics information through single-cell next-generation sequencing (NGS), migration assays, RNA-Seq, and EPISPOT immunoassays^77–79^. Perhaps the most intriguing potential applications of CTCs are personalized functional assays using patient CTC xenografts in mice or *in vitro* cultures^80^. Such an assay has already been used to assess the efficacy of drugs in prostate cancer patients, with assay results corresponding well with patient drug resistance status^81^.

Unlike CTCs, ctDNA does not require specialized sample preparation steps for detection and can often be detected in samples in which CTCs are absent^79,82^. ctDNA is likely released by apoptotic or necrotic cells within a tumor, or by the destruction of CTCs via apoptosis, the immune system, or anoikis^77,82,83^. Like CTCs, increased levels of ctDNA are generally associated with later stage disease or disease recurrence after treatment. The two primary types of information that can be gleaned from ctDNA are mutation status and methylation status, though a limited degree of copy number variation analysis may also be possible. ctDNA mutations can be assessed by a variety of techniques including allele-specific polymerase chain reaction (PCR), digital PCR, and tagged-amplicon deep sequencing (TAm-Seq)^82^. ctDNA mutation status could play a key role in monitoring disease progression during treatment and checking for the presence of drug-resistant subclones^74,76^. Methylation status is typically assessed with methylation specific PCR and can be used to reliably distinguish ctDNA from nontumor derived cell-free DNA. Major challenges that ctDNA diagnostics face include lack of standardization (such as how many mutations constitute a “positive” result when used for screening), potentially confounding mutations due to clonal expansion of benign cells, and difficulty in establishing personalized assays for the general population, that is, those without an established history or risk of cancer^76^.

#### Organoids

One approach currently under development for personalized treatment of cancers is patient-derived tumor organoids, which serve as *in vitro* tumor models and predictors of drug responses^84^. Traditional approaches to cancer research and therapies involve the use of *in vitro* cancer cell lines, patient-derived xenografts, and 3D culture models. These are limited by their inability to accurately correlate an individual tumor’s response to a treatment due to the diversity and heterogeneity of the tumor microenvironment. Organoids offer a more accurate representation of this dynamic niche and there is evidence that the genomic and functional resemblances between patient-derived tumor organoids and their original specimens can be nearly identical^85–88^. The original success of tumor organoid cultures came from Weeber and colleagues, who successfully reported 90% preservation of somatic mutations and DNA copy number profile between the developed tumor organoids and patient original biopsies. This was achieved across a total of 1,977 cancer-related genes from 14 patients with metastatic colorectal cancer^85^. Other positive developments in the use of these organoid models were reported by van de Wetering *et al.*^86^ The group successfully established a biobank of 20 colorectal carcinoma (CRC) derived tumor organoid cultures. Each culture represented a major CRC mutation subtype that was confirmed by whole-exome sequencing analysis. This allowed for a more accurate detection of gene–drug associations for each individualized subtype of CRC. Another promising study conducted an examination of drug sensitivities of tumor-derived organoids against a library of 63 drugs in 232 treatment regimens^89^. Tumors resected from 14 patients with refractory advanced cancers were propagated in mice and treated. Researchers were successfully able to identify an effective treatment for 12 of the 13 individual patients in the xenograft model. Therefore, 11 of the 12 patients received their prospectively guided treatments, with one patient having died before treatment. This data supports the use of personalized xenograft models for guided treatment platforms. Tumor-derived organoids provide a means for an accurate representation of gene–drug association on an individual basis, with the ease-of-use of an *in vitro* model. Hence, organoids hold immense potential to play significant roles in the development of PPM cancer therapies.

#### Targeted mAbs for cancer therapy

Out of the many molecular-based techniques (e.g., small molecules, mAbs, and vaccines), mAbs have been very promising for cancer therapeutics due to their low cytotoxicity, high specificity, and scalability^90–92^. mAbs are Y-shaped proteins, produced either synthetically or by B lymphocytes, that have the ability to bind to a specific molecular target. mAbs are one of the fastest growing immunotherapies; there are over 22 FDA approved mAbs-based drugs for oncology.

In contrast to traditional therapies (e.g., surgery, radiotherapy, and/ or chemotherapy), therapies based on mAbs are targeted to specific molecular markers that a particular tumor expresses, and are therefore likely to be more effective. For instance, human epidermal growth factor receptor 2 (HER2) positive breast cancers result in better clinical benefits from HER2-targeted mAbs (e.g., trastuzumab and pertuzumab) than mAbs that target HER2 negative breast cancer markers (e.g., everolimus)^93^. Additionally, epidermal growth factor receptor (EGFR) mAbs are commonly used for treatments of KRAS wild-type colorectal tumors, but nearly half of treated patients have not shown any clinical benefits^94^. Interestingly, under some conditions, tumors even continue to mutate and develop primary resistance against the targeted molecule^95^. Ultimately, the choice of the mAb (or the combination of mAbs) will often be defined by the cancer type, cancer subtype, and overall efficacy and side effects from other clinical and preliminary studies.

Recent advances in NGSTs at the single-cell level have provided researchers with more precise information about novel drug targets. This work has improved mAbs that target specific antigens on cancer cells and resulted in a more personalized approach^96–99^. Merck’s pembrolizumab (Keytruda®) became the first drug to target a genetic signature (biomarker PD-L1 expressed in 50% of the non-small cell lung cancer) rather than a disease^100^. In a Phase III clinical trial, treating patients with pembrolizumab, combined with a first line chemotherapy drug, resulted in a 36% higher response rate and lower side effects compared to treating patients with only chemotherapy^101^. Recently, mAbs in combination with other mAbs or chemotherapy have entered mainstream targeted cancer therapy. In addition to cancer, mAb therapies are also used to treat autoimmune diseases, infection, and hematological diseases. With increasing demand for PPM, current projections reveal that the global mAb therapy market is projected to grow to approximately $1.5 trillion by 2021 and would account for about 20% of biopharmaceutical market share^102^.

#### Immune checkpoint inhibitors

A promising advancement in cancer treatment is the development of antibodies capable of blocking coinhibitory immune cell receptors, or “immune checkpoints” — T-cell surface receptors that, when activated by particular ligands, reduce the T-cell’s cytotoxic immune response. Tumor cells tend to overexpress the ligands that activate these inhibitory receptors, thereby evading the T-cell immune response and proliferating freely^103^. Though over two dozen different costimulatory receptors have been identified^104^, two — CTLA-4 and programmed cell death 1 (PD-1) — have been the focus for antibody-based immune checkpoint blockade (ICB) treatments, and six such drugs have been approved by the FDA^105^. CTLA-4 was the first identified negative regulator of T-cell activity^106^; when activated, it delivers inhibitory signals blocking T-cell proliferation and secretion of T-cell maturation agent IL-2^107^. The CTLA-4 inhibitor ipilimumab became the first FDA-approved ICB drug in 2011 after a clinical trial demonstrated its beneficial impact on survival rates in stage III and IV melanoma patients^108^. PD-1 was identified as a coinhibitory T-cell receptor in 1999^109^ and, unlike CTLA-4, represses T-cell activity primarily by promoting T-cell exhaustion^110^. The first PD-1 targeting ICB drug, nivolumab, was approved by the FDA in 2014, following favorable outcomes compared to chemotherapy in a clinical study administering nivolumab to patients whose melanoma progressed after ipilimumab treatment^111^. Since then, nivolumab has received FDA approval as a first-line treatment in non-small-cell lung cancer^112^, renal-cell carcinoma^113^, urothelial carcinoma^114^, Hodgkin’s lymphoma^115^, and more. A second key anti-PD-1 ICB drug, pembrozilumab, has FDA-approval for similar treatments and also recently became the first anticancer drug to receive “site-agnostic” approval — it is cleared for use on all mismatch-repair deficient solid cancers, regardless of tissue type, with particular biomarkers^116^. The newest FDA-approved ICB drugs, including atezolizumab^117^ and durvalumab^118^, target PD-L1, the PD-1 ligand, thereby providing the same inhibition of PD-1 activation via a different chemical approach. Combinatory approaches involving simultaneous use of both CTLA-4 and PD-1 or PD-L1 inhibitors^119^, or PD-1 inhibitors with additional T-cell costimulators^120^, are currently under development with promising preliminary results.

In the context of PPM, effectively using these therapies will require diagnostics to determine the likelihood of a particular patient’s tumor responding appropriately to the ICB drug. Further investigations into the cellular mechanisms of the immune checkpoint are undergoing, with the aim of identifying biomarkers and other diagnostic features that could predict a patient’s response to this immunotherapy^121^. Potential biomarkers for anti-PD-1-based therapies include direct assessment of PD-L1 expression, density of tumor-infiltrating lymphocytes, and quantity of mutation-related neoantigens in tumor cells; effective treatment will likely require using a combination of these and unknown other markers^122^. The frequency of CD4 T-cells expressing the inducible costimulator (ICOS) marker has been found to be a robust pharmacodynamic biomarker for anti-CTLA-4-based treatment efficacy^123^. Development of clinical tests using these and other potential markers will enable a personalized immunotherapy approach for a wide variety of solid cancers.

#### Cancer vaccines

Cancer vaccines, which have long been envisaged as effective tools for cancer immunotherapy, are designed to amplify the tumor-specific T-cell response through active immunization^124^. Through selection of a suitable antigen target present on tumor cells, a potent and tumor-specific immune response can be induced. Studies have shown that tumor neoantigens, or antigens encoded by tumor-specific mutated genes, have a key role in therapeutic vaccination. Recent efforts in acquiring omics data through NGS have allowed for the systematic discovery of tumor neoantigens that arise from somatic mutations and are therefore, tumor-specific^124^. This specificity allows for diverse tumor neoepitopes (“peptides that arise from somatic mutations and are recognized as different from self”^125^) between individuals. Identifying these candidate tumor neoantigens on a per-patient basis has led to the development of personalized cancer vaccines^124^. RNA-Seq data from thousands of samples across 18 different solid tumors from The Cancer Genome Atlas demonstrated a positive correlation between the number of neoantigens per tumor type and T-cell cytolytic activity specific for those tumors^124^. Furthermore, whole-exome sequencing analysis of 629 colorectal cancers showed that high neoantigen loads are associated with improved patient survival due to the ability to target multiple neoantigens at one time^126^. Preclinical experiments in both a melanoma model and a transplantable colon cancer model revealed that neoantigen vaccination elicited a selective T-cell response and effectively mediated antitumor activity. In a cholangiocarcinoma patient, adoptive transfer of neoantigen-specific CD4^+^ T-cells mediated tumor regression, demonstrating the clinical success of this therapy^127^. Therefore, the concept of targeting multiple neoantigens as a personalized cancer vaccine strategy has been realized and is being further researched and developed.

There is currently only one FDA-approved cancer vaccine, sipuleucel-T, which is indicated for metastatic prostate cancer that no longer responds to hormonal therapy^128,129^. It is based on the use of dendritic cells taken from the patient’s blood. In the lab, the dendritic cells are treated with prostatic acid phosphatase (PAP), an antigen that is found on most prostate cancer cells. Antigen-presentation is enhanced, so when the dendritic cells are infused back into the patient, their T-cells react by killing PAP-expressing tumor cells.

#### CAR T-cell therapies

Genetically engineered CAR T-cell therapies have also shown great promise in the advancement of individualized cancer immunotherapies^130^. Autologous T-cells are engineered to express a CAR that specifically targets and kills malignant cells or can be directed to remodel the tumor microenvironment through release of soluble factors^130^. Through recent advances in NGSTs, treatments that target tumor niches with a high degree of specificity can be adapted to account for the tumor microenvironment’s heterogeneity and complexity. Gathering large data sets that describe different tumor phenotypes/genotypes provides the possibility of precise, individualized design, and optimization of CAR T-cell-based therapies^131^. Current advancements in genome editing, including CRISPR and gene transfer, have improved CAR T-cell therapy development by increasing their tumor-specificity^130^.

CAR T-cell therapies exemplify a personalized approach to cancer therapy because they directly prime a patient’s cells to better combat their own cancer. Thus far, this has been most successful in patients with relapsed or refractory malignancies who are resistant to treatment, particularly in chronic lymphocytic leukemia (CLL), which remains incurable through conventional therapies^132^. Results from initial trials using CAR-modified T-cells to treat 14 patients with CLL showed 8 out of 14 (57%) successful responses with 4 complete remissions and 4 partial remissions with no relapses. Other successful preclinical and clinical trial data have led to the two first FDA-approved genetically engineered cell therapies. Both are CAR T-cell products, Kymriah and Yescarta, which treat patients with relapsed or refractory B-cell acute lymphoblastic leukemia (ALL) and nonresponsive B-cell lymphoma, respectively. The safety and efficacy of Kymriah was demonstrated in 63 pediatric and young adult patients with ALL with overall remission rate within three months being 83%^133^. Unfortunately, like with many biologics and gene therapies, Kymriah has proven to show variability in manufacturing, limiting its market availability^134^. However, with continued advancements in these cell therapy technologies, the ability to tailor each individual patient’s treatment for their particular cancer is an attainable goal in the near future.

### Companion diagnostics

CDx are medical devices that aid doctors in prescribing the most effective, personalized treatments for their patients^18^. Relevant genetic information for characterizing cancers is found in defined stretches of DNA (i.e., oncogenes). In order to avoid sequencing the entire genome and obtaining extraneous information, some CDx are based off these specific oncogenes and can be used to determine whether or not a person will respond to a certain treatment. Each CDx is associated with a particular drug therapeutic, which, in turn, is associated with a specific genetic abnormality for which it is most effective^18^.

Gaining insight into the molecular makeup of each patient’s cancer eliminates the misuse of ineffective and potentially harmful drugs. Studies on cancer and tumor heterogeneity have led to the discovery of various genetic mutations known to drive cancer progression, for example, HER2 mutations in the case of some breast cancers^135–137^. This discovery led to the development of therapeutics to target these precise mutations such as trastuzumab (Herceptin), which is the first approved precision therapeutic to combat breast cancer caused by overexpression of the HER2 gene^136,138,139^_._

A variety of diagnostic methods exist within the category of CDx products, each serving a specific functionality. These include immunohistochemistry (IHC), fluorescent *in situ* hybridization (FISH), and RT-qPCR (Table 5)^140^. Table 5 is a current overview from the FDA.gov website of the existing FDA approved CDx devices used in oncology. Many companies have developed different CDx devices specifically for trastuzumab, as this drug has been approved by the FDA since 1998^136^.

It is interesting to note that not all CDx play the role of identifying patients that would benefit from a given therapy, as in the case of the FDA-approved CDx QIAGEN Therascreen^141^. This RT-qPCR type diagnostic is used to eliminate patients from receiving the drugs Vectibix and Erbitux for metastatic colorectal cancer. The Therascreen PCR kit is meant to detect seven different mutations in the KRAS gene. When patients suffer from a highly mutated form of colorectal cancer, they will no longer benefit from taking Vectibix or Erbitux. Therefore, the doctor will not prescribe them to these patients, preventing the use of unnecessary and ineffective medications that come with negative side effects.

### Regulations for PPM

**Figure 4:**
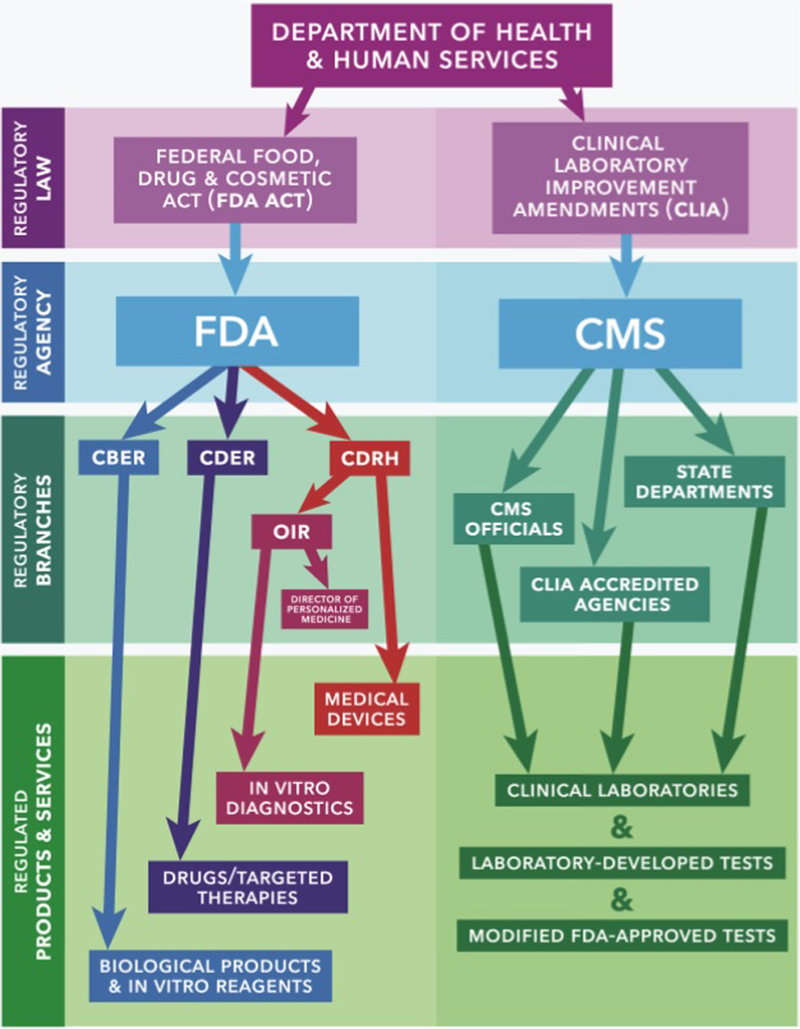
Regulatory landscape for PPM products and services. A look at the structure of the agencies responsible for regulating PPM products. The FDA is responsible for evaluating the safety and efficacy of all medical devices, pharmaceutical products, and biological products sold in the United States. Most CDx tests and treatment products fall under FDA jurisdiction. The CMS oversees all clinical laboratories in the United States, certifying that they meet quality and proficiency standards for collecting and interpreting clinical data. Generally, the CMS is responsible for approving laboratory-developed diagnostic tests.

As the technological race advances and new tests and treatments that target specific patient populations are developed, regulatory agencies must devise novel approaches to ensure the safety, efficacy, and security of these products while allowing for innovation^142,143^. The regulatory landscape has been changing quickly, due in part by the enactment of the Precision Medicine Initiative in 2015, which required the FDA to develop a new platform to evaluate new PPM diagnostics and therapies^19^, and the 21st Century Act (Cures Act) in 2016, which accelerated medical product development by incorporating the patients’ perspective and also modernized clinical trial design^144^. The resulting evolution of the regulatory paradigm has driven an increase in the number of FDA approved PPM products and services. In 2005, only 5% of new drug approvals were PPMs, however, in 2017, over 30% of new drug approvals (16 new therapies) were PPMs^9,145,146^. The development of regulations that have allowed PPM to enter the market has involved several different agencies, guidance documents, and approaches (Fig. 4).

#### Regulatory agencies overseeing PPM products and services

The FDA and the Center for Medicare and Medicaid Services (CMS), both falling under the Department of Health and Human Services (HHS), are the two agencies that hold primary responsibility for overseeing PPM services and products used in clinics, laboratories, and hospitals around the country^147^.

All medical devices, pharmaceutical products, and biological products sold in the United States are evaluated by the FDA for safety and efficacy before entrance to market, using a risk-based approach^142^. Different centers within the FDA regulate different types of medical products^147^, as depicted in Fig. 4. These centers are involved with the approval and oversight of all products that fall into their defi ning categories, and therefore, also oversee relevant PPM products.

Regulations that fall outside of the FDA’s jurisdiction belong to CMS, CMS-approved third-party organizations, and state programs, which oversee rules pertaining to all clinical laboratories in the United States through the Clinical Laboratory Improvement Amendments (CLIA)^148^. CMS certifies that labs meet and maintain certain standards before performing tests and interpreting results on human samples. CLIA requirements generally include qualifications for laboratory personnel, quality systems for lab testing, oversight of test requests and reports, and proficiency testing^147^.

#### Types of PPM and associated regulations

A wealth of products, innovations, and tests fall under the umbrella of PPM, and therefore, regulatory agencies must consider the appropriate requirements suited for each type. In this review, the discussion will be limited to CDx, NGS-based diagnostic tests, and laboratory developed tests (LDTs).

##### CDx regulations.

As defined by the FDA, a CDx is “a medical device, often an *in vitro* device, which provides information that is essential for the safe and effective use of a corresponding drug or biological product”^149^. CDx assist healthcare providers in determining if a product’s benefits outweigh its risks for patients.

Since CDx are recognized as medical devices by the FDA, they are subjected to the premarket review process. The FDA recommends that a therapeutic product and its accompanying diagnostic test be developed and submitted for approval at the same time; if not, there is a risk of delaying the introduction of the product to the market and limiting access to patients. For example, Herceptin and HercepTest, the first therapeutic product and CDx combination cleared by the FDA, were approved 6 months apart. Although this time gap was relatively short, it was recognized as a potential future risk for products if not developed together^9^. As a result, the FDA has since released two guidance documents: a final guidance in 2014 titled *In Vitro Companion Diagnostic Devices Guidance*, which helped clarify its method for conducting simultaneous reviews of a therapeutic product and its associated CDx; and a draft guidance in 2016 titled *Principles for Codevelopment of an In Vitro Companion Diagnostic Device with a Therapeutic Product*, which explained how therapeutic and diagnostic partners should interact with the FDA when codeveloping combination products. In addition, the FDA has recognized that routine biomarker testing prior to prescribing certain drugs is a class of CDx that will continue to grow. The FDA has therefore begun compiling a table of genomic biomarkers that they consider valid in guiding the clinical use of approved drugs^150^.

##### *In vitro* diagnostics — regulations for next-generation sequencing tests and laboratory-developed tests.

The FDA defines an *in vitro* diagnostic (IVD) as a “test to identify patients who are likely to benefit from specific treatments or therapies”^151^. IVDs may be marketed in one of two ways: as IVD kits or as LDTs, which present another set of challenges for regulatory agencies. The main difference between IVD kits and LDTs is that IVD kits are developed by a conventional device manufacturer and are commercially available for healthcare providers, while LDTs are designed, manufactured, and used by a single laboratory^147^. As a consequence of this distinction, the regulatory jurisdiction of IVD kits and LDTs has generally fallen into two separate agencies, the FDA and CMS, respectively. In addition, if an FDA-approved IVD kit is modified by a clinical laboratory, it will be classified as an LDT (falling into CMS jurisdiction) and will not be required to undergo premarket review; however, if the same IVD kit is modified by a device manufacturer, it will be subjected to the premarket review process (FDA jurisdiction)^152^. This dichotomy has created more confusion about the proper regulatory path for new PPM products. The approaches that the FDA has taken to ensure the safety and reliability of IVDs are described below.

One type of IVD is NGS-based tests, which are used to find genetic variants that help diagnose, treat, and understand more about human disease^151^. The thorough sequencing capabilities of NGSTs present a challenge for the current regulatory approaches, which were developed for conventional diagnostics that detect a single disease or condition. In contrast, a single NGS test can yield the equivalent amount of information that millions of traditional tests provide^143^. Therefore, NGS test development and regulation of NGS-based IVD will require more flexible oversight, which the FDA has pursued by using consensus standards, crowd-sourced data, and open-source computing technology approaches^143^. According to the FDA, “this strategy will enable innovation in testing and research, and will expedite access to accurate, reliable genetic tests”^151^.

In an effort to streamline the regulatory oversight of NGS-based tests by leveraging crowd-sourced data and consensus standards, the FDA released two final guidance documents in 2018: *Use of Public Human Genetic Variant Databases to Support Clinical Validity for Genetic and Genomic-Based In Vitro Diagnostics*, which describes the process of developing and using FDA-recognized public genome databases to support the clinical validity of a test, and *Considerations for Design, Development, and Analytical Validation of Next Generation Sequencing (NGS) — Based In Vitro Diagnostics (IVDs) Intended to Aid in the Diagnosis of Suspected Germline Diseases*, which provides recommendations for designing, developing and validating NGS-based tests^143^. In addition, it encourages the development of NGS-related standards by community engagement and standards-developing organizations. Furthermore, the FDA has developed a bioinformatics platform named *precisionFDA*. This is an open-source cloud-based community that allows individuals and organizations in the genomic field across the world to share data and tools to test, pilot, and validate bioinformatics approaches^143,153^. This platform further enhances the widespread collaboration that is needed for the technological development of NGS-based tests and demonstrates the FDA’s support of this notion as they work to create suitable regulations.

LDTs, which are diagnostic tests that are designed, manufactured, and used within a single laboratory, also fall under the broad category of IVDs^147,154^. Since these tests are made for “in-house use” and are not commercially distributed, their regulatory oversight has generally fallen under CMS jurisdiction, which subjects them to CLIA rules. Although the FDA has claimed authority to regulate LDTs, it has generally chosen not to actively exert this power under the “enforcement discretion” policy^147^. This policy has been historically applied to simple LDTs, such as in-house vitamin D or sodium assays; however, LDTs have since become more complex and therefore pose higher risks for the patient — risks that are similar to those associated with other IVDs regulated by the FDA^154^. This change in the nature of LDTs, with specific regards to PPM, has led the FDA to occasionally exert its power. This confusion and current lack of a regulatory path for LDTs has made it unclear in which specific cases FDA requirements also apply in addition to those from CMS. For example, in 2005 the FDA subjected the MammaPrint (Agendia BV) breast cancer recurrence assay to premarket approval. The lack of data showing clinical benefits to patients was a major concern for the overseeing FDA officials. Several years later, in 2008, MammaPrint finally received FDA approval, when the markers proved clinical benefits for patients with breast cancer. The MammaPrint assay was reclassified as an *in vitro* diagnostic multivariate index assay (IVDMIA), which is a type of LDT^147^. As seen by this example, the FDA has exercised regulatory authority over LDTs to varying degrees under different circumstances. This, in combination with several other factors (e.g., FDA vs. CMS oversight, categorization of IVDs as medical devices vs. LDTs, and different guidance documents/standards applied), has led to confusion and uncertainty in the market, which has hindered biotechnology and pharmaceutical industries’ investment in the PPM field.

Due to the FDA’s evolving concerns regarding the rapid expansion and the intended uses of certain LDTs as CDx for PPM products and services, the agency issued two draft guidance documents in 2014 titled *Framework for Regulatory Oversight of Laboratory Developed Tests (LDTs)* and *FDA Notification and Medical Device Reporting for Laboratory Developed Tests (LDTs)*. The goal of these documents was to provide clarity regarding the extent of FDA oversight of LDTs. However, after engaging with multiple stakeholders and revising more than 300 comment sets and alternative proposals, the agency recently announced that it will not yet issue final guidance documents on this topic^154^. This decision was made to allow for further public discussion, and hopefully consensus, on an appropriate regulatory approach. Instead, the FDA published a discussion paper in 2017 summarizing the feedback received and alternative proposals to further advance public discussion on LDT oversight^152^.

#### Future regulatory landscape for PPM

Despite the regulatory challenges that exist, the processes outlined by several guidance documents (Table 6) reflect the FDA’s willingness to adapt to the changing landscape of medicine^9^, along with consideration of feedback from scientists, clinicians, and patients. In response to the increase in the number of PPM products and services, the growing demand for regulatory clarity, and the enactment of the Precision Medicine Initiative and the Cures Act, the FDA began working on the PPM platform over a decade ago. Its aim is to provide a rapidly evolving strategy to approve new PPM diagnostics and drugs, while maintaining high standards of safety and efficacy. Nevertheless, the regulatory landscape of the PPM field is still emerging — and is still convoluted — due to the complex nature of many PPM products and services that fall under the oversight of multiple regulatory centers. Moreover, the vast data sets that are generated from some PPM products, particularly NGS-based tests, present large challenges for regulatory agencies, as privacy concerns must also be considered. As PPM becomes an even larger part of modern medicine, it is pertinent for discussions regarding regulations to be on-going and for regulatory documents to be continually adapted and updated. Based on recent changes to how the FDA will be changing regulations governing gene therapy in order to streamline review, the agency recognizes the need for these adaptations^155^. Efforts that address difficult regulatory decisions regarding PPM may begin to cover other controversial topics surrounding this field, particularly in regards to economics and ethics.

## BROADER CONSEQUENCES OF PPM

Thus far in this review, we have considered the science and technology behind PPM — the sequencing, the data analysis, and the development of CDx — but what about the broader implications of PPM on health-care? When it comes down to cost, is PPM worth the investment for biopharmaceutical companies? Is it worth the investment in the eyes of healthcare insurers? And, is it an unnecessary risk to acquire vast amounts of sensitive and personal health information that can potentially be used against patients? Here we discuss important considerations that must be made as PPM quickly enters the clinic and reaches more patients.

### Economics

In 2015, national healthcare spending in the United States was $3.2 trillion, or $9,900 per person, with $324.6 billion spent on prescription drugs^156^. This makes the United States the largest healthcare spender in the world. Despite being the leader in healthcare spending, Americans have poor health outcomes, including shorter life expectancy and greater prevalence of chronic conditions, when compared to 12 other high-income countries (Australia, Canada, Denmark, France, Germany, Japan, Netherlands, New Zealand, Norway, Sweden, Switzerland, and the United Kingdom)^157^. Many proponents of PPM believe it has the ability to reduce healthcare spending through the identification of therapy responders and nonresponders during both clinical trials and, upon approval, in clinical use. This section seeks to explore how PPM can be used in both drug development and clinical use and if healthcare costs will be reduced, both for payers and patients.

#### PPM in drug development

The cost to develop a drug, taking it from the laboratory bench top to market, currently exceeds $2.5 billion^157^. Developing new therapies is a high-risk, expensive, and long-term endeavor. Moreover, the number of successful candidates is incredibly small, and those few drugs that do make it to market must support the development costs of all other drugs in the pipeline. Because costs are so high, pharmaceutical companies pass expenses to the consumers. Companies need to recoup the money invested in order to fund the research and development for the next generation of therapies.

PPM has the potential to reduce the risk and cost of drug development, particularly in clinical trials, one of the most expensive stages of development. The cost savings are rooted in stratifying patients into smaller subsets and identifying a population that is more likely to respond well to the proposed therapy, oftentimes with the use of CDx. By focusing on smaller populations, clinical trial size will shrink, substantially reducing the costs. In addition, the population admitted to the trial is more likely to respond to the therapy, reducing the risk associated with failed clinical trials^138^.

The cost savings and reduced risk of clinical trials associated with the PPM has been quantified for a number of diseases. Studies have shown that approximately 11% of drugs that enter Phase I clinical trials obtain FDA approval. However, clinical trials of targeted therapies have higher success rates. Falconi *et al.* conducted an analysis of stage IIIb–IV clinical trials of non-small-cell lung cancer therapies. In the 676 analyzed trials that occurred between 1998 and January 2012, biomarker targeted therapies had a 62% cumulative success rate. This is almost six times greater than the 11% cumulative success rate for any drug entering a Phase I clinical trial. Further, they found therapies that targeted receptors provided the largest cumulative success rate of 31% when compared to other therapeutic mechanisms. These results suggest that through the use of biomarkers and PPM, there are therapeutic mechanisms and design strategies that can decrease the amount of risk during drug development^158^. The Falconi *et al.* study quantified the reduction in risk-adjusted drug development costs. The cost for stage IIIb–IV non-small-cell lung cancer was estimated to be $1.9 billion. However, the use of a biomarker in this disease treatment resulted in a 26% reduction in risk-adjusted drug development costs.

The cost and risk reduction found for non-small-cell lung cancer are consistent with findings from the analyses of trials for other diseases. Parker *et al.* analyzed trials for advanced metastatic breast cancer that stratified patients that were positive for the HER2 biomarker compared to patients that had either failed or had been exposed to anthracycline or taxane^159^. The overall success rate of new drug development in anthracycline/taxane-exposed patients was only 15%, while in the HER2-positive patients it was 23%. The cost for the clinical trial testing alone, when adjusted for risk, was $199 million for the HER2-positive patients, substantially lower than the $274 million for the anthracycline/taxane patients. This represents a 27% cost savings and reduced clinical trial risk up to 50%. Parker *et al*. also published analyses of non-Hodgkin’s lymphoma clinical trials, again confirming targeted therapies had a higher success rate versus nontargeted, broad acting therapeutics^160^. While limited to the oncology field and a small number of cancer types, these analyses suggest that a PPM approach can lead to significant cost savings and risk reduction during the drug development and clinical trial process. More retrospective cost and risk analyses of clinical trials must be completed, as the benefit of this approach is likely to vary greatly in different disease types.

#### Reducing patient cost with PPM

In traditional patient care, when a patient is presented with a specific indication, the doctor will prescribe a first-line therapy^161^. Generally, the physician does not take into account patient demographics or disease-specific biomarkers when prescribing this therapy. If the first-line treatment does not work, the physician will try a second-line treatment or use a combinatorial approach to treat the patient. This approach does not identify a patient’s likelihood of positive response to treatment, nor does it predict if the patient will have severe side effects. In the United States, the cost of adverse drug reactions in 2013 was more than $30 billion^162^. Through the use of diagnostics to stratify patients into responders, nonresponders, and those likely to have severe side effects, this cost can be reduced^163^.

In the current healthcare landscape, PPM approaches are used only after other therapies fail. Particularly in oncology, these conventional treatments, such as radiation and chemotherapy, can take an enormous toll on patients, leaving them exhausted, weakened, and unprepared for later treatments. In some cases, it limits patients’ abilities to travel to clinical trial sites to participate in potentially lifesaving studies^161^. Worse, it can take a great deal of time to determine if these first-line treatments are having a positive effect — time that patients with advanced conditions rarely have.

When PPM approaches are finally employed, large portions of the cost of these tests and therapeutics typically fall to the patients. This is in addition to the costs they have already incurred during first line therapies. Medicare and other payers frequently classify genomic-based screening and treatment into specialty tiers which require patients to pay amounts that far outweigh typical copays^161^. Often, patients are expected to assume a minimum of 20%–40% of the total cost of treatment. With these specialized approaches and complex biologic therapeutics, it is common for these costs to reach tens of thousands of dollars and even higher. This can put PPM treatments out of financial feasibility for some, and the patients and loved ones who do receive treatment can be saddled with crippling hospital expenses.

There are several omics approaches that can be used to identify the best therapy for a patient. Certainly, the identification of disease-specific genetic variants and biomarkers that can be exploited is an important undertaking, but the enormity of the work, time, and cost involved should not be understated. Furthermore, the costs of performing genetic analysis for every patient ahead of first line therapies is currently unrealistic, despite the fact that technology continues to lower sequencing costs. Another promising approach is using metabolomics to determine how a patient will metabolize specific therapeutics. This approach is more direct and efficient in providing meaningful information compared to other omics approaches, since many pharmaceuticals are metabolized by just a few proteins in the liver and many adverse drug reactions can be traced back to variations in these enzymes. Brixner *et al.* demonstrated this approach in a 2016 study that tested elderly patients for genetic variations in cytochromes P450, a family that contains major enzymes involved in drug metabolism^164^. The study followed patients whose treatment was informed using a medication management clinical decision support tool. It was observed that the patients that were DNA tested and treated according to the personalized prescribing system had significantly lower hospitalizations and emergency department visits, resulting in cost savings. These results are consistent with a previous study that tested patients for known drug–gene interaction risk to inform their treatment protocol^165^.

As the aforementioned studies have demonstrated, identifying responders or nonresponders can result in cost savings during both drug development and clinical use of pharmaceuticals. While this potential savings is promising, the real question is if healthcare spending will actually be decreased through the use of these tools. Many supporters say yes, but skeptics point out that by stratifying patients and targeting therapies, the pharmaceutical companies are shrinking their available market share, which could result in a price increase to offset reduced volume. This means that this strategy is only economically beneficial in markets with pricing flexibility, where buyers and sellers are able to negotiate on price. Pricing flexibility will vary based on payer’s price sensitivity, which is highly dependent on disease area. Not only is pricing flexibility a requirement to make this approach economically attractive, but the underlying pathophysiological principles of the targeted disease must be understood. This leaves conditions like psychiatric disorders, which carry a great societal and healthcare burden, out of the current scope of PPM, as they are currently too poorly understood to benefit from the approach^163^.

#### Adoption by payers

While PPM may hold great potential to reduce costs in the long run, it is unclear who foots the bill in the interim. Currently there are very few instances of private insurance companies or government payers providing reimbursement for broad-based genomic testing and analysis. Insurance companies and payers rely on mountains of outcome-based data to determine what they will cover. Payers are hesitant to support new and untested treatments without overwhelming evidence that they will be effective. As PPM is a new and emerging field, a sufficient level of data and evidence has not been accumulated to support widespread reimbursement for such treatments in the eyes of the payers.

The amount of evidence is growing, however, and some payers are catching on. In December 2015, Foundation Medicine announced that they had reached an agreement with UnitedHealthcare, one of the largest private insurance companies in the United States, on their genomic profiling assay^166,167^. UnitedHealthcare agreed to reimburse the use of the genomic profiling assay for patients with metastatic stage IV non-small-cell lung cancer. This move has been described as a critical first step toward bringing genomic profiling into the standard treatment of care for metastatic cancers. While it is a positive move toward wider adoption, both in terms of indications and payers, the greatest industry shift toward PPM will likely come when Medicare adopts a reimbursement policy for PPM treatments. Medicare represents the largest single payer in the United States, comprising 20% of total national health expenditures in 2015^156,168^. As such, many private insurance companies use Medicare to benchmark their own coverages. When and if Medicare adds increased coverages for broad genomic assays as a part of standard treatment, other insurance companies would surely follow.

On the surface, a PPM approach holds great potential benefit to both the pharmaceutical industry and patients. However, with the complexities of the existing healthcare environment, involving drug developers, regulators, clinicians, and payers, the immediate and lasting benefits are not as clear. Trends toward outcome- and value-based pricing and reimbursement models greatly increase the financial value of PPM. This type of model will require collaboration between regulatory agencies and industry to develop and shape adjusted drug development and approval processes. Additionally, collaboration and willingness of payers to adopt these approaches is critical to make PPM economically viable and beneficial for patients and the industry as a whole.

### Ethics

Tied into the economic considerations of PPM are the ethical considerations. With the added power of harnessing large amounts of medical data comes the heavy responsibility of protecting and distributing it correctly. The medical field is now poised to move from a “one size fits all” approach to a PPM method of treating patients based on the individuality of this information. However, this comes at a cost, as some patients may be at a disadvantage due to a shift in allocation of resources throughout the healthcare system. There are also issues related to implementation and control of data. Clinicians may have to reach an agreement with insurance companies and researchers that allows for providing the best possible treatment while also protecting patient privacy.

Generating genetic information and linking it to patient outcome will aid researchers and clinicians immensely, but it also means that the practice of informed consent will need to be substantially updated^169^. Some have suggested the development of “translational ethics” that involves the patients as much as possible in the research and clinical processes. One successful study, CARPEM, formed a patient committee group tasked with designing a pamphlet explaining the informed consent process in a patient-friendly manner^170^. Others have suggested updating the “social contract” between clinicians, researchers, patients, and society as a whole^171^. Since patients are the stakeholders who will benefit the most from advances in PPM, involving them as much as possible may be the best strategy for moving forward.

This review has focused on cancer for the sake of brevity, but it is easy to imagine how similar issues would apply to psychiatric disorders or autoimmune diseases. Regardless of the clinical application, as the field of PPM advances, standard ethical practices surrounding clinical medicine and research will need to be updated.

## OUTLOOK

Overall, the promise of PPM is exciting and inspiring, and it has the potential to transform the way in which cancer is effectively treated. As a result of PPM, if omics testing is performed prior to treatment, patients would be less likely to experience adverse side effects from a treatment that is ultimately ineffective, for example, chemotherapy, and, rather, would only spend time and resources on personalized, effective treatments. The incentive for pharmaceutical industries would be to develop more effective drugs that have a greater chance of being approved, albeit for a smaller population^172^. This shift toward a more tailored treatment experience would benefit patients to a great extent in the long run. Making this a reality, however, requires many key players — physicians, insurance companies, and regulators — to come together for the benefit of the patient. These players each have their own compelling reasons for resisting this transition to PPM, including needing enough evidence to support new strategies or concern about the profitability of disease prevention. Regulators are caught between physicians, insurers, and pharmaceutical companies and must decide which agencies are responsible for modifying the rules in the new era of PPM. Ultimately, it may be up to patients to push for these changes since they stand to benefit the most^172^.

While it is important to recognize the promise and potential of PPM, it is also important to ask whether the lofty goals proposed by advocates are realistic. Will we actually be able to treat each patient individually? The answer is almost certainly not, but we may be able to treat subpopulations more effectively. Moreover, depending on how the costs to cover the implementation of PPM are distributed, who will truly benefit from it? The answer is, in the short term, probably only those with private insurance and enough disposable income will be able to afford additional genetic tests^172^. Additionally, even if healthcare based on PPM was equally accessible to all, it might not reach those who need it most. Instead of focusing on improving clinical care, it has been posited that we should be working to remedy the social structures that prevent disadvantaged groups from leading healthier lives. This may in fact be the best way to improve population health since income is one of the most significant determinants of health outcomes^173^.

## SUMMARY AND CONCLUSIONS

The PPM field has grown and matured tremendously since the milestone achievement of sequencing an entire human genome in 2003. Research has moved beyond sequencing more accurately to linking this information to individual patient outcomes and treatment responses. Many challenges still remain in sorting through massive quantities of biological data to identify clinically relevant markers for disease susceptibility and treatment efficacy. Cancer treatment in particular stands to highly benefit from PPM therapies, since extensive variability between tumors presents a need to target each case in a personalized manner. Recent work has focused on the development of more accurate tumor models (organoids) and harnessing the specificity of the immune system to develop effective cancer vaccines or mAbs.

The personalized treatment approach has resulted in improved patient outcomes in terms of response rate and progression-free survival in Phase I clinical trials that selected patients using a specific biomarker versus those that did not^174^. The improvements between the personalized versus nonspecific approach were 30.6% versus 4.9% response rate and 5.7 versus 2.95 months progression-free survival in cancer patients. These statistics show a dramatic improvement in patient response when they are matched to treatments for their specific disease; however, there is still much room for improvement. Additionally, development of PPM therapies must be performed with careful regards to evolving regulations. As researchers acquire PPM data and companies develop PPM therapies, regulators, clinicians, patients, and the public must consider the broader consequences of PPM. A major collaborative effort between all associated groups — scientists, biopharmaceutical companies, insurers, clinicians, regulators, and patients — will be necessary to keep driving PPM forward and make it a viable field that benefits all.

## Figures and Tables

**Table 1:** Comparison of next generation sequencing technologies. A summary of next-generation sequencing technologies, which are used to collect genomics data. Different clinical applications require technologies with different advantages, so consideration of accuracy, cost, time, throughput, and ease of use is required before selecting a sequencing technology for clinical use.

Technology	Description	Applications	Run time^175^	Max. reads length^175^	Max. read per run^175^	Approx. cost^a^	Accuracy^175^	Advantage(s)	Disadvantage(s)
Sanger Sequencing	First-generation sequencing technique, contemporarily useful for verifying NGS sequences, if needed. Foundation for current NGS techniques^176^	Applicable for small sequencing reads	20 min–3 hr	400–900 bps	1,000	$5–20	99.99%	Accurate Low read length Shorter time	Low throughput High cost per run
Illumina MiSeq	Instrument capable of sequencing 96 samples in single run. Detects fluorescence emitted after synthesis of DNA strands with sample templates^177,178^	Small genome sequencing	4–55 hr	2 × 150 bps	25 million	$700–1,500	98%	High throughput	High read length
		Targeted gene, miRNA, and small RNA profiling							
		16S metagenomic sequencing							
Illumina NextSeq	Fluorescence-based multiplexed sequencing Instruments tailored for specific applications^179^	Nextseq: Small whole-genome for microbe or virus; exome, and transcriptome sequencing	12–30 hr	2 × 150 bps	400 million	$1,000–5,000	98%	High throughput	High read length
Illumina HiSeq		HiSeq: Exome and whole-transcriptome sequencing	1 hr–6 days	2 × 150 bps	5 billion	$1,000–4,000	98%	High throughput	High read length
Illumina NovaSeq 6000		NovaSeq: Large whole-genome sequencing for animals and plants; exome and whole-transcriptome sequencing; methylation sequencing	16–44 hr	2 × 150 bps	20 billion	$2,000–5,000	98%	High throughput	High read length
SOLiD	Highly accurate fluorescence-based method^180^	Small genome sequencing	7–14 days	2 × 50 bps	1.5 billion	$5,000–10,000	99.94%	Accurate	High read length
Ion Torrent	System detects pH change resulting from H^+^ release in solution of growing DNA chain corresponding to sample template^23^	Small genome sequencing	2 hr	200 bps	15 million	$200–500	99%	More stable with longer reads	High read length
SMRT from Pacific Bioscience	Fluorescence-based multiplexed method noteworthy for its use of the world’s smallest light detection volume for the site of sequencing. This minimizes noise of fluorescence readings^181^	Small genome sequencing	4–6 hr	1,300 bps	89 million	$300–500	97%	Does not require PCR amplification during sample prep	Low throughput

a Cost — subject to change based on the facility and the discount.

Abbreviations: bps: base pairs, NGS: next generation sequencing, PCR: polymerase chain reaction.

**Table 2:** Overview of transcriptome assessment tools. The transcriptome is the set of RNA molecules expressed from a patient’s cancerous cells. Microarray analysis and RNA-Seq are the two major ways to collect transcriptomics data. Generally, RNA-Seq is performed in exploratory studies, which attempt to identify RNA sequences linked to cancer phenotypes. Once these sequences are known, microarray analysis is performed on patient samples to determine which sequences are present.

Strategy	Purpose	Data collection	Data analysis
Microarray analysis	samples for expression abundance. Requires prior knowledge of sought RNA sequences^25,26^	RNA is reverse-transcribed to double stranded cDNA which is then fragmented and fluorescently labeled^26^. Probes are short oligonucleotides that hybridize to fluorescent cDNA fragments^25^	Fluorescence intensity indicates the abundance of gene expression. Computational image analysis allows for quantification^25^
RNA-Seq	Analysis of high-throughput RNA samples for expression abundance and sequence discovery. Sequence discovery tool — does not require prior knowledge of RNA sequence^26^	RNA is fragmented then reverse-transcribed to ds cDNA. cDNA is amplified via PCR to yield the RNA-Seq library, used as a reference genome. Iteratively, fluorescently labeled nucleotide bases are washed over the library, binding to nucleotides in order of their sequence. Fluorescence is captured in each iteration, preserving the order of the sequence^25,26^	Fluorescence images preserve sequence order and abundance of mRNA sequences. Indicates mRNA expression abundance in sample. Computational image analysis allows for quantification^25,26^

Abbreviations: PCR: polymerase chain reaction, RNA-Seq: RNA sequencing.

**Table 3:** Overview of proteomics strategies and workflows. A summary of strategies for obtaining proteomic data — information on the protein species present in a patient’s cancerous tissues. Typically, bottom-up or “shotgun” approaches are used in exploratory studies to identify proteins that are linked to particular cancer phenotypes. Top-down and middle-down strategies are more useful for analyzing samples from patients. In characterizing cancer for PPM treatments, identification of post-translational modifications and other high-level protein features is especially important, as these features are valuable targets for PPM therapies.

Strategy	Purpose	Data collection	Data analysis
Bottom-up (shotgun) proteomics	Analysis of large mixed protein samples and determination of their composition, e.g., in biomarker discovery studies	Proteins are broken into peptides through trypsin proteolysis and the peptides are separated based on size or charge in a mass spectrometer^182^	Mass spectra are compared to a database like Andromeda^183^ or PeptideAtlas ^184^ for protein identification
Bottom-up proteomics with labeling	Enables simultaneous multiple-sample analysis of proteomic changes, e.g., changes due to biological perturbations	Isotopes of C, H, N, and O added to peptide samples via methods such as SILAC, ICAT, and iTRAQ^185^, and peptides are analyzed via tandem MS (MS/MS)	Relative peptide abundances are measured by comparing intensities of the different isotope species in the MS/MS data
Top-down proteomics	Analysis of whole proteins, with special interest in post-translational modifications	Proteins are ionized and converted to gas stage using techniques such as MALDI and ESI^186^, then separated using LC and analyzed via MS	Mass spectra are compared to protein databases, such as ProSight PTM which offers a free Windows app for sequence identification^187^
Middle-down proteomics	Produces less complex solutions for easier protein identification and analysis of high-level protein features^188^	Proteins are digested only enough to produce large peptide fragments, which are analyzed via MS	MS analysis identifies both protein composition and the presence of high-level features such as protein isoforms and modifications^32^

Abbreviations: ESI: electrospray ionization, ICAT: isotope-coded affinity tags, iTRAQ: isobaric tags for relative and absolute quantitation, LC: liquid chromatography, MALDI: matrix-assisted laser desorption/ionization, MS: mass spectrometry, PPM: precision and personalized medicine, SILAC: stable isotope labeling with amino acids in cell culture.

**Table 4:** Overview of metabolomics strategies and workflows. Like the other omics data collection approaches, metabolomics data collection can be summarized with two main strategies, untargeted and targeted. The untargeted approach is used in exploratory studies to link metabolite profiles to cancer phenotypes, and targeted metabolomics is used to analyze samples from patients to determine which metabolites are present. Metabolomics is a relatively new field and its application in PPM is just beginning.

Strategy	Purpose	Data collection	Data analysis
Untargeted (global) metabolomics	Analysis of large mixed metabolite samples and determination of their composition, e.g., in biomarker discovery studies	Metabolites are isolated using LC or techniques, such as solvent-dependent precipitation^189^, polarity and ionization filtration^190^, and quenching with methanol^191^, then quantified using MS/MS	Mass spectra are compared with MS/ MS databases, which include Scripps’ METLIN^192^, the Human Metabolome Database^193^, and MassBank^194^
Targeted metabolomics	Quantify known metabolites in a particular sample, e.g., to analyze a patient’s condition	Metabolites of interest are separated from the sample using a variety of common separation techniques^195^, and measured using MS/MS	Mass spectra are compared with calibration curves based on measuring known amounts of the metabolites of interest

Abbreviations: LC: liquid chromatography, MS: mass spectrometry, PPM: precision and personalized medicine.

**Table 5:** FDA-approved CDx for cancer treatments, by company. The FDA is responsible for evaluating the safety and efficacy of all medical devices, pharmaceutical products, and biological products sold in the United States (see Fig. 4). This table lists FDA-approved CDx for cancer treatment. Each of these products is used to detect a particular omics feature that is linked to a specific cancer phenotype. Positive results from these diagnostic tools help to indicate the potential efficacy of a PPM treatment.

Device manufacturer	CDx name	Drug	Type	Disease	Device/Test specifics
Abbott Molecular	VYSIS ALK Break Apart FISH Probe Kit	Crizotinib	FISH	NSCLC	Detect rearrangements in the ALK gene in fixed NSCLC tissue from patients with NSCLC
Abbott Molecular	PATHVYSION HER-2 DNA Probe Kit	Trastuzumab	FISH	Breast cancer	Detect amplification of HER2/NEU gene in fixed, breast cancer tissue samples. Aid in predicting disease-free and overall survival in patients with stage II, node positive breast cancer treated with adjuvant cyclophosphamide, doxorubicin, and 5-fluorouracil (CAF) chemotherapy
Abbott Molecular	Abbott Real Time IDH2	Enasidenib	PCR	AML	Detects single nucleotide variants coding nine IDH2 mutation in samples extracted from patient’s blood or bone marrow
Abbott Molecular	VYSIS CLL FISH Probe Kit	Venetoclax	FISH	CLL	Detect deletion of the LSI TP53 probe target from peripheral blood samples from patient with B-cell CLL
ARUP Labs	PDGFRB FISH	Imatinib mesylate	FISH	MDS/MPD	Qualitative detection of PDGFRB gene rearrangement from fresh bone marrow samples of MDS/MPD patients
ARUP Labs	KIT D816V Mutation Detection	Imatinib mesylate	PCR	ASM	Qualitatively determines the mutation level of the KIT D816V gene via fresh bone marrow samples of ASM patients
BioGenex Labs	INSITE HER-2/NEU Kit	Trastuzumab	IHC	Breast cancer	Semiquantitatively determine the overexpression of HER-2/ Neu of fixed normal and neoplastic breast cancer tissue sections
bioMérieux	THxID BRAF Kit	Trametinib and dabrafenib	PCR	Melanoma	Detection of either BRAF V600E or BRAF V600K mutations in DNA samples extracted from fixed, melanoma tissue. Patients who carry V600E mutations are eligible for dabrafenib and those who carry V600K mutations are eligible for trametinib
Dako Denmark	HERCEPTEST	Trastuzumab, pertuzumab, and ado-trastuzumab emtansine	IHC	Breast and gastric cancer	Determine HER2 protein overexpression in fixed breast cancer, metastatic gastric, or gastroesophageal junction adenocarcinoma tissues
Dako Denmark	HER2 FISH PharmDx Kit	Trastuzumab, pertuzumab, and ado-trastuzumab emtansine	FISH	Breast and Gastric cancer	Quantitatively determine HER2 gene overexpression in fixed breast, metastatic gastric, or gastroesophageal junction adenocarcinoma tissues
Dako Denmark	HER2 CISH PharmDx Kit	Trastuzumab	FISH	Breast cancer	Quantitatively determine HER2 gene status of fixed, breast cancer tissue specimens
Dako North America	DAKO EGFR PharmDx Kit	Erbitux and vectibix	IHC	Colorectal cancer	Identify EGFR expression in both fixed, normal and neoplastic tissue samples from patient
Dako North America	DAKO C-Kit PharmDx	Imatinib mesylate	IHC	GIST	Qualitative measure to identify c-kit protein/CD 117 antigen expression in both fixed normal and neoplastic tissue samples
Dako North America	PD-L1 IHC 22C3 pharmDX	Pembrolizumab	IHC	NSCLC	Using EnVision FLEX visualization system to detect PD-L1 protein in fixed, NSCLC samples
Foundation Medicine	FoundationOne CDx^a^	Numerous	PCR	Numerous	Detects: substitutions, insertions, deletions and copy number alterations in 324 genes, select gene rearrangements, genomic signatures such as microsatellite instability and tumor mutational burden, from patient tissue biopsies
Foundation Medicine	FoundationFocus CDxBRCA Assay	Rucaparib	PCR	Ovarian cancer	NGS-based detection of BRCA1 and BRCA2 (BRCA1/2) alterations from fixed, ovarian tissue samples
Illumina Inc.	Praxis Extended RAS Panel	Panitumumab	PCR	Colorectal cancer	Detects 56 mutations in RAS genes from DNA extracted from patient tissue samples
Invivoscribe	LeukoStrat CDx FLT3 Mutation Assay	Midostaurin	PCR	AML	Detects internal tandem mutations and the tyrosine kinase domain mutations D835 and I836 in FLT3 gene from mononuclear cell DNA of AML patients
Leica Biosystems	Bond Oracle HER2 IHC System	Trastuzumab	IHC	Breast cancer	Semi-Quantitative assay to determine HER2 protein levels of fixed, breast cancer tissues using the bond-max slide staining instrument
Life Technologies	Oncomine Dx Target Test	Dabrafenib, trametinib, crizotinib, and gefitinib	PCR	NSCLC	Detects single nucleotide variants and deletions in 23 genes from DNA and fusions in ROS1 from RNA, isolated from patient tumor tissue samples
Life Technologies	SPOT-LIGHT HER2 CISH Kit	Trastuzumab	FISH	Breast cancer	Quantitatively determine HER2 gene overexpression from fixed breast carcinoma tissues using CISH and brightfield microscopy
MolecularMD Corporation	MolecularMD MRDx BCR-ABL Test	Nilotinib	PCR	CML	Detection of BCR-ABL1 transcripts and the ABL1 endogenous control mRNA in patient blood samples whom are receiving treatment for tyrosine kinase inhibitors
Myriad Genetic Labs	BRACAnalysis CDx	Olaparib	PCR	Ovarian cancer	Detection and classification of DNA variants in the protein coding regions and intron/exon boundaries of BRCA1/2 genes using whole blood samples from patients
QIAGEN Manchester	Therascreen EGFR RGQ PCR Kit	Afatinib	PCR	NSCLC	Detection of exon 19 deletions and exon 21 (L858R) substitution mutations of EGFR gene from fixed, NSCLC tissue
QIAGEN Manchester	Therascreen KRAS RGQ PCR Kit	Cetuximab and panitumumab	PCR	Colorectal cancer	Detection of seven somatic mutations in codons 12 and 13 of the KRAS gene in fixed, colorectal cancer tissue. Treatment of erbitux and vectibix is issued upon a NO mutation test result
QIAGEN Manchester	Therascreen EGFR RGQ PCR Kit	Gefitinib	PCR	NSCLC	Detection of exon 19 deletions and exon 21 (L858R) substitution mutations of EGFR gene from fixed, NSCLC tissue
Roche Molecular Systems	The COBAS KRAS Mutation Test	Cetuximab and panitumumab	PCR	Colorectal cancer	Detection of seven somatic mutations in codons 12 and 13 of the KRAS gene in fixed, colorectal cancer tissue. Treatment of erbitux and vectibix is issued upon a NO mutation test result
Roche Molecular Systems	COBAS EGFR Mutation Test	Erlotinib	PCR	NSCLC	Detect deletion of exon 19 and substitution mutations of exon 21 (L858R) of EGFR gene in DNA from fixed NSCLC tissue
Roche Molecular Systems	COBAS EGFR Mutation Test v2	Erlotinib	PCR	NSCLC	Qualitative detection of defined mutations of the EGFR gene in NSCLC patients. Test can be run using fixed NSCLC tissue samples or circulating free tumor DNA
Roche Molecular Systems	COBAS EGFR Mutation Test v2	Osimertinib	PCR	NSCLC	Detect T790M mutation of EGFR gene in DNA of fixed NSCLC tissue or ctDNA from NSCLC patients
Roche Molecular Systems	COBAS 4800 BRAF V600 Mutation Test	Vemurafenib	PCR	Melanoma	Qualitative detection of BRAF V600E mutation in DNA extracted from fixed melanoma tissue from patient
Ventana Medical Systems	VENTANA ALK (D5F5) CDx Assay	Crizotinib	IHC	NSCLC	Intended for the detection of ALK in fixed NSCLC tissue stained with a BenchMark XT instrument
Ventana Medical Systems	INFORM HER-2/NEU	Trastuzumab	FISH	Breast cancer	Determines the qualitative presence of HER2/NEU gene amplification from fixed, breast cancer tissue samples
Ventana Medical Systems	INFORM HER2 DUAL ISH DNA Probe Cocktail	Trastuzumab	FISH	Breast cancer	Determine HER2 gene status via enumeration of the ratio of the HER2 gene to chromosome 17 using fixed, breast cancer tissue from patient
Ventana Medical Systems	PATHWAY ANTI-HER-2/NEU (4B5) Rabbit Monoclonal Primary Antibody	Trastuzumab	IHC	Breast cancer	Semiquantitative detection of c-erbB-2 antigen (HER2) in fixed, breast cancer tissue specimens using the Ventana automated IHC slide staining device
Ventana Medical Systems	PD-L1	Atezolizumab	IHC	Urothelial carcinoma and NSCLC	Assess PD-L1 protein expression levels in fixed, patient tissue samples (stained with OptiView DAB IHC Detection Kit and OptiView Amplification Kit on a VENTANA BenchMark ULTRA instrument)

a First FDA-approved CDx for a broad spectrum of applications.

Abbreviations: ALK: anaplastic lymphoma kinase, ASM: aggressive systemic mastocytosis, BRCA: breast cancer susceptibility gene, CDx: companion diagnostics, CISH: chromogenic *in situ* hybridization, CLL: chronic lymphocyctic leukemia, CML: chronic myeloid leukemia, EFGR: epidermal growth factor receptor, GIST: gastrointestinal stromal tumors, FDA: Food and Drug Administration, FISH: fluorescence *in situ* hybridization, FLT3: FMS like tyrosine kinase 3, HER2: human epidermal growth factor receptor 2, IDH2: isocitrate dehydrogenase 2, IHC: immunohistochemistry, MDS/MPD: myelodysplastic syndrome/myeloproliferative disease, NSCLC: non-small-cell lung cancer, PCR: polymerase chain reaction, PDGFRB: platelet derived growth factor receptor beta, PD-L1: programmed death-ligand 1, PPM: precision and personalized medicine.

*Source:* Information compiled and modified from U.S. Food and Drug Administration^140^.

**Table 6:** FDA policy and guidance documents on PPM. A summary of guidance documents developed by the FDA related to PPM regulation and oversight. Regulatory processes for PPM products, which often encompass multiple FDA categories, are complex, but the FDA has been willing to adapt to the continual evolution of PPM treatments as evidenced by the publication of these standards.

Year	Guidance document	Status
2005	Pharmacogenomic Data Submissions	Final guidance^a^
2007	Pharmacogenomic Tests and Genetic Tests for Heritable Markers	Final guidance
2007	*In Vitro* Diagnostic Multivariate Index Assays	Draft guidance
2008	E15 Definitions for Genomic Biomarkers, Pharmacogenomics, Pharmacogenetics, Genomic Data, and Sample Coding Categories	Final guidance
2011	E16 Guidance on Biomarkers Related to Drug or Biotechnology Product Development: Context, Structure, and Format of Qualifications Submissions	Final guidance
2012	Enrichment Strategies for Clinical Trials to Support Approval of Human Drugs and Biological Products	Draft guidance
2013	Clinical Pharmacogenomics: Premarket Evaluation in Early-Phase Clinical Studies and Recommendations for Labeling	Final guidance
2013	Clinical Pharmacogenomics: Premarket Evaluation in Early-Phase Clinical Studies and Recommendations for Labeling	Final guidance
2014	Qualification Process for Drug Development Tools	Final guidance
2014	*In Vitro* Companion Diagnostic Devices	Final guidance
2014	Framework for Regulatory Oversight of Laboratory Developed Tests (LDTs)	Draft guidance
2014	FDA Notification and Medical Device Reporting for Laboratory Developed Tests (LDTs)	Draft guidance
2016	Use of Standards in FDA Regulatory Oversight of Next Generation Sequencing (NGS)-Based *In Vitro* Diagnostics (IVDs) Used for Diagnosing Germline Diseases	Draft guidance
2016	Use of Public Human Genetic Variant Databases to Support Clinical Validity for Next Generation Sequencing (NGS)-Based *In Vitro* Diagnostics	Draft guidance
2016	Principles for Codevelopment of an *In Vitro* Companion Diagnostic Device with a Therapeutic Product	Draft guidance
2017	Discussion Paper on Laboratory Developed Tests (LDTs)	Discussion paper (no enacted guidance)
2018	Use of Public Human Genetic Variant Databases to Support Clinical Validity for Genetic and Genomic-Based *In Vitro* Diagnostics	Final guidance
2018	Considerations for Design, Development, and Analytical Validation of Next Generation Sequencing (NGS) — Based *In Vitro* Diagnostics (IVDs) Intended to Aid in the Diagnosis of Suspected Germline Diseases	Final guidance

a Note that guidance documents provide insight into FDA’s policies, but are not legally binding.

FDA: Food and Drug Administration, PPM: precision and personalized medicine.

*Source:* Adapted from Personalized Medicine Coalition^9^.
